# Cellulose ether treatment inhibits amyloid beta aggregation, neuroinflammation and cognitive deficits in transgenic mouse model of Alzheimer’s disease

**DOI:** 10.1186/s12974-023-02858-y

**Published:** 2023-07-28

**Authors:** Tahir Ali, Antonia N. Klein, Keegan McDonald, Lovisa Johansson, Priyanka Ganguli Mukherjee, Martin Hallbeck, Katsumi Doh-ura, Hermann M. Schatzl, Sabine Gilch

**Affiliations:** 1 Calgary Prion Research Unit, Faculty of Veterinary Medicine, University of Calgary, 3330 Hospital Drive NW, Calgary, AB T2N 4Z6 Canada; 2 Hotchkiss Brain Institute, University of Calgary, Calgary, AB Canada; 3 Department of Biomedical and Clinical Sciences (BKV), Linköping University, 58185 Linköping, Sweden; 4 Microscopy Imaging Facility (MIF), University of Calgary, Calgary, AB Canada; 5 Department of Neurochemistry, Tohoku University Graduate School of Medicine, Sendai, Miyagi Japan

**Keywords:** FDA-approved cellulose ethers, Alzheimer’s disease (AD), Astrogliosis, Microgliosis, Glial maturation factor beta, Memory functions, Neuroinflammation

## Abstract

Alzheimer’s disease (AD) is an incurable, progressive and devastating neurodegenerative disease. Pathogenesis of AD is associated with the aggregation and accumulation of amyloid beta (Aβ), a major neurotoxic mediator that triggers neuroinflammation and memory impairment. Recently, we found that cellulose ether compounds (CEs) have beneficial effects against prion diseases by inhibiting protein misfolding and replication of prions, which share their replication mechanism with Aβ. CEs are FDA-approved safe additives in foods and pharmaceuticals. Herein, for the first time we determined the therapeutic effects of the representative CE (TC-5RW) in AD using in vitro and in vivo models. Our in vitro studies showed that TC-5RW inhibits Aβ aggregation, as well as neurotoxicity and immunoreactivity in Aβ-exposed human and murine neuroblastoma cells. In in vivo studies, for the first time we observed that single and weekly TC-5RW administration, respectively, improved memory functions of transgenic 5XFAD mouse model of AD. We further demonstrate that TC-5RW treatment of 5XFAD mice significantly inhibited Aβ oligomer and plaque burden and its associated neuroinflammation via regulating astrogliosis, microgliosis and proinflammatory mediator glial maturation factor beta (GMFβ). Additionally, we determined that TC-5RW reduced lipopolysaccharide-induced activated gliosis and GMFβ in vitro. In conclusion, our results demonstrate that CEs have therapeutic effects against Aβ pathologies and cognitive impairments, and direct, potent anti-inflammatory activity to rescue neuroinflammation. Therefore, these FDA-approved compounds are effective candidates for developing therapeutics for AD and related neurodegenerative diseases associated with protein misfolding.

**Supplementary Information:**

The online version contains supplementary material available at 10.1186/s12974-023-02858-y.

## Introduction

Alzheimer’s disease (AD) is a progressive, devastating, and incurable neurodegenerative disorder that is clinically characterized by gradual loss of learning and memory functions. AD is the most common type of dementia. The neuropathological hallmark of AD includes the accumulation of misfolded proteins, such as amyloid beta (Aβ) that forms extracellular senile plaques, and intracellular neurofibrillary tangles of hyperphosphorylated tau proteins in the cortex, hippocampus and other brain regions [1–4]. Misfolded Aβ accumulation precedes neurofibrillary tangle formation [5]. Aβ oligomers (AβO) and Aβ fibrils induce AD pathologies, causing oxidative stress, aberrant neurotransmission, neuroinflammation, misfolding of other proteins and consequently, lead to synaptic and neuronal damage, which triggers cognitive deficit. Aβ is generated by the cleavage of a transmembrane amyloid precursor protein (APP) by beta-site amyloid precursor protein cleaving enzyme 1/beta-site APP cleaving enzyme 1 (BACE-1) and γ-secretase [5–11]. Aβ aggregation triggers the activation of numerous proinflammatory mediators in activated glial cells [12] leading to neuroinflammation. It is widely reported that chronic neuroinflammation in turn generates further Aβ accumulation, creating a vicious cycle, which leads to the progression of AD pathologies. Neuroinflammation also plays a vital role in the pathogenesis of AD and is critically involved in the progression of brain degeneration [13–15]. Hence, it is important to target both misfolded Aβ and subsequent neuroinflammation to halt and treat AD.

In this study, our primary objectives were to explore the therapeutic efficacy of cellulose ether (CE)-derived compounds for the treatment of AD. Numerous studies reported the health benefits of dietary supplementation of CE-derived hydroxypropyl methylcellulose (HPMC), which improves peripheral metabolic disorders in animals, including humans [16–26]. The US Food and Drug Administration (FDA) approved HPMC as a safe pharmaceutical and food additive and reported that quantities up to 670 mg in oral formulations are safe to humans [27–29]. However, recently our and other groups’ studies showed that CEs have therapeutic effects in neurodegenerative diseases via inhibiting the propagation of prions, infectious agents consisting of a misfolded protein [30–35]. Most importantly, subcutaneous (SC) administration of CEs even one year prior to prion infection significantly increased the life span of prion-infected mice [31]. Prions share their mechanism of propagation with other misfolded proteins including Aβ and α-synuclein. Moreover, recent studies extended the CE effect in prion diseases and demonstrated that it is dependent upon immune cells along with the proinflammatory glial maturation factor beta (GMFβ) gene and other genes which are in its vicinity [36, 37]. Several studies reported that GMFβ has been found to be upregulated in several neuroinflammation and neurodegeneration conditions including AD and Parkinson’s disease [38–51]. The beneficial effect of CEs in prion diseases and findings demonstrating that CEs inhibit amyloid formation by crowding effects highlight the potential beneficial effects of CEs in the treatment of multiple neurodegenerative diseases associated with protein misfolding and amyloid formation [52–54]. Therefore, in this study we aimed to determine the therapeutic efficacy of CEs in both in vitro and in vivo AD models. We found that TC-5RW, a HPMC representative, inhibited Aβ aggregation, reduced Aβ neurotoxicity and rescued its associated pathologies such as activation of proinflammatory marker GMFβ and activated gliosis in transgenic 5XFAD mice overexpressing human APP and presenilin 1 (PSEN1) transgenes harboring in total 5 mutations associated with familial AD. Of note, for the first time we demonstrate that CE treatment significantly improves learning and memory functions in the 5XFAD mouse model of early-onset, familial AD. Overall, our findings suggest that CE treatment targets both Aβ aggregation and neuroinflammation, breaking the vicious cycle leading to clinical AD.

## Materials and methods

### Mouse strains, housing, and animal ethics statement

The transgenic hemizygous 5XFAD mice (B6SJL-Tg (APPSwFlLon, PSEN1*M146 L*L286 V)6799Vas/Mmjax) and wild type (WT) control mice with the same genetic background were purchased from Jackson Laboratory, USA. The mice were housed at 12-h (h)/12-h light/dark cycle and a maintained temperature at 23 °C, in an environment with 60 ± 10% humidity. The mice were allowed to access food and water ad libitum. All experiments related to mice in this study were approved by the University of Calgary Health Sciences Animal Care Committee (AC18-0030) according to the guidelines issued by the Canadian Council for Animal Care and international ARRIVE guidelines.

### Single and chronic administration of TC-5RW to 5XFAD mice

For the first experiment, 6-week-old 5XFAD female mice were divided into two groups the non-treated 5XFAD control group (*n* = 5 mice) and a group of 5XFAD (*n* = 10 mice) treated with a single dose of TC-5RW (4 g/kg) subcutaneously (SC) [31–33]. TC-5RW was dissolved in ultra-pure de-ionized distilled water at 37 °C for 1 h. After complete solubilization, TC-5RW was infused very slowly and carefully SC at the neck of mice. At the age of 10 months, mice were euthanized using 5% isoflurane and cervical dislocation. The brains were extracted for biochemical and immunohistochemical analysis.

In the second experiment, we used male mice at the age of 6 weeks and included four groups: (1) control WT, 10 male mice; (2) control 5XFAD, 15 male mice; (3) 5XFAD mice (15 male) received a weekly SC dose of TC-5RW (4 g/kg; WI) started at the age of 6 week and continued till the age of 9 months; (4) 5XFAD mice (15 male) were SC injected with a single dose of TC-5RW (4 g/kg; SI), at the age of 6 week and we performed behavioral experiments at the age of 9–10 months of all four groups, i.e., WT, 5XFAD, 5XFAD (WI) and 5XFAD (SI).

### Behavioral assessments of mice

To assess the cognitive functions of mice, we performed the novel object recognition (NOR), Y-maze and contextual fear conditioning (FC) tests.

### NOR test

A white square plastic box (approx. 40 × 40 cm) and 2 sets of different items (each set has varied in texture, shape, and color) were used for NOR test. Mice were habituated by gentle handling for 2 days prior to training and testing sessions. In the training session, we kept two identical objects with the same distance from each corner and side of the box. All mice were placed in the same place in the box and allowed to freely explore two identical objects for 5 min and then they were returned to their home cage. In the testing session on the next day, mice were allowed to explore one familiar and one novel object of different shape and texture but similar height with the familiar object. The time to explore both familiar and novel object was recorded. The recognition index (RI) was calculated as the percentage of time spent by mice exploring the novel object divided by the total time spent to explore both objects.

### Y-maze test

The Y-maze test evaluates mice’ short-term spatial learning and memory behavior. The Y-maze consists of three equally spaced arms labeled with letters A, B and C. Each mouse was placed at the end of one arm for 8 min and allowed to move freely to enter another arm. Entries into each arm were recorded and alternation behavior was defined as entry into all three arms sequentially. Spontaneous alteration was defined as the successive entry of the mice into the three arms in overlapping triplet sets. Alteration behavior (%) was calculated as follows: [successive triplet sets (entries into three different arms consecutively)/total number of arm entries-2] × 100.

### FC test

After completion of NOR and Y-maze tests, we performed FC test using a chamber with plastic walls and a metal mesh floor. The inner dimensions of the chamber are approximately 17 cm × 17 cm × 25 cm. Before starting the training and testing, mice were acclimatized for 1–2 h in the behavioral rooms. After acclimatization, on day one mice were placed into the conditioning chamber and habituated to their surroundings for at least 2 min. Following habituation, the mice received three pairings of tone/light signals (20 s, 80 dB; or user specific setting) and a co-terminating electric shock (1 s, 0.5 mA). The inter-trial interval between each of the pairings was 2 min. Once the trial was complete, the mice were returned to their home cage. The chamber was cleaned with 70% ethanol after each mouse. On the final testing day, each animal was then placed in the same chamber for 6 min (testing). The tone/light in the absence of shocks was presented twice 20, 120, and 260 s after the animal was placed into the chamber. During this testing period, the behavior of the mouse was recorded by a digital video camera directly mounted above the conditioning chamber. The amount of time spent freezing is quantified and defined by the complete absence of motion. FC was conducted with the ANY-maze (Stoelting Co. UK). Freezing detection is automatically quantified by the software with the default freezing detection settings.

### Extraction of mouse brains and preparation of brain homogenates

Brain hemispheres were separated, and homogenates were prepared according to our previously published protocol [55]. Briefly, one brain hemisphere was homogenized in 0.1 M phosphate buffered saline (PBS) (10% w/v) using a gentle MACS™ Dissociator for 2 min at room temperature, followed by centrifugation at 2000*g* for 1 min at 4 °C. The homogenates were aliquoted and stored at − 80 °C until further processing for immunoblotting, dot blotting and other biochemical assays.

### SDS‑PAGE (sodium dodecyl sulfate-polyacrylamide gel electrophoresis) and immunoblotting

Ten % brain hemisphere homogenates were mixed with equal volume of cold protein extraction buffer, while cell lysates were mixed with the relevant amount of cold protein extraction buffer according to the number of cells and according to manufacture protocol (PROP-PREP™, Catalogue number: 17081, iNtron Biotechnology, USA). Protein samples were prepared in 1 × SDS sample buffer and processed on 12.5–18% Tris–HCl SDS-PAGE gel. However, for separation of low molecular weight Aβ, samples were loaded on pre-cast tris–tricine SDS-PAGE gels (ThermoFisher Scientific, USA) or prepared according to previously published method [56].

Electroblotting was done using Amersham Hybond P 0.45 µm and 0.22 µm PVDF membranes (Amersham, USA). After blocking with 5% nonfat dry milk in 1× TBST, membranes were incubated with primary antibodies at 4 °C overnight. Membranes were washed three times each for 5 min and then incubated in secondary antibodies according to the source of primary antibodies at room temperature at least for 1 h. After washing at least three times each for 5 min, membranes were developed using Luminata Western Chemiluminescent HRP Substrates (Millipore, USA). ImageJ was used for densitometric analysis of immunoblots.

### Enzyme-linked immunosorbent assay (ELISA) for soluble and insoluble Aβ42

The soluble and insoluble Aβ42 levels were analyzed in brain homogenates from 5 and 5XFAD mice treated with TC-5RW. The ELISA was performed according to the manufacturer’s protocols (Invitrogen, Thermo Fisher Scientific, Rockford, IL, USA). Briefly, for the soluble and insoluble Aβ42 analyses we used 10% brain homogenates in cold protein extraction buffer and spun for 15–25 min at 10,000 rpm and then removed the supernatant for the soluble Aβ42 contents. Similarly, for insoluble Aβ42 the remaining pellet was incubated in 70% formic acid for 30–40 min and following the formic acid incubation the suspension was spun for 15–25 min at 10,000 rpm and collected the supernatant for the measurement of insoluble Aβ42 contents. Soluble and insoluble Aβ42 results were analyzed and calculated as pg/ml.

### Collection of mouse brain sections for immunohistochemical analyses

Mice were anesthetized deeply with isoflurane (5%) and continued control anesthesia at 2.5% isoflurane while mice were transcardially perfused with 0.1 M PBS. The mouse brain hemispheres were post-fixed in 4% paraformaldehyde for 72 h and brain tissues were cryoprotected with 20% sucrose for 72 h in 0.1 M PBS at 4 °C for 3–5 days until they completely sank. Brain hemispheres were frozen in OCT (optimum cutting temperature) compound (A.O, USA), and 12-μm coronal sections were collected using a CM 3050C cryostat (Leica, Germany). Brain tissue sections were thaw-mounted on commercially available ProbeOn Plus charged slides (Fisher, USA).

### Immunofluorescence staining and confocal microscopy

Single and double-immunofluorescence staining was performed as described previously with some modifications [55, 57]. Briefly, gelatin-coated slides containing brain tissues were dried 2–4 h at room temperature. After drying, the slides were washed twice for 5 min each in 0.01 M PBS. Following washing, the tissues were processed for antigen retrieval by incubation for 10 min at room temperature in 1× proteinase-k. The slides were washed twice for 5 min each, followed by incubation for 1 h in blocking solution containing 2% FBS and 0.1% Triton X-100 in 0.01 M PBS according to the source of primary antibodies. After blocking, tissue sections were incubated overnight at 4 °C in the primary antibodies (Table 1), washed twice for 5 min each and incubated for at least 1 h with donkey anti-mouse IgG H&L (FITC) (ab6816, Abcam, USA), rhodamine (TRITC) donkey anti-goat IgG (H + L) Alexa Fluor 488, goat anti-rabbit, or Alexa Fluor™ 555 goat anti-rabbit secondary antibodies (Jackson Immunoresearch, USA) (1:100). For double-immunofluorescence, primary and secondary antibody incubations were repeated (Table 1). Then slides were washed twice for 5 min each and coverslips were mounted with DAPI along with Dako fluorescent mounting medium (Molecular Probe, Eugene, OR). All images were captured under the same conditions using a confocal laser scanning microscope (Zeiss LSM 700). Several images per section (tissue) and more than 20 images per field of each brain area were captured from each respective group. Confocal images were converted to tagged image file format (TIF) and quantification of immunofluorescence intensity in the same region of the brain areas was performed using ImageJ software. The background of TIF images was optimized according to the threshold intensity. The immunofluorescence intensity was analyzed at a specified threshold intensity for all groups under the same conditions and was expressed as the relative integrated density between the groups.

**Table 1:** List of primary antibodies and their detailed information

Antibody name	Host species	Application	Manufacturer	Catalog/reference number	Concentrations
Aβ (6E10)	Mouse	WB/DB/IF	Biolegend, USA	803001	1:2000/1:100
Aβ (4G8)	Mouse	WB/DB/IF	Biolegend, USA	800708	1:2000/1:100
GMFβ	Rabbit	WB/IF	ProteinTech, USA	10690-1-AP	1:2000/1:100
GMFβ	Rabbit	WB/IF	Abcam, USA	Ab224322	1:2000/1:100
Aβ (B4)	Mouse	WB/DB/IF	Santa Cruz Biotechnology, USA	SC-28365	1:1000/1:100
GFAP	Mouse	WB/IF	=	SC: 33673	1:40,000/1000
Iba-1	Mouse	WB	=	SC: 32725	1:1000
Iba-1	Goat	IF	Novus Biologicals, LLC, USA	NB100-1028	1:25
GFAP	Rabbit	IF	=	BN300-141	1:1500
β-Actin	Mouse	WB	Sigma Aldrich, USA	A5441	1:40,000

*WB* western blotting, *IF *immunofluorescence, *DB* dot blotting

### Preparation of amyloid beta monomer (AβM), AβO and Aβ fibrils for in vitro experiments

Aβ1-42 peptide was oligomerized as previously described [58, 59] with few modifications. Briefly, recombinant Aβ1-42 (Innovagen) was dissolved in 1,1,1,3,3,3-hexafluoro-2-propanol (HFIP) (Sigma Aldrich, USA), and lyophilized using a Savant Speed Vac Plus freeze drier (ThermoFisher, USA). The peptides were then resuspended in dimethyl sulfoxide (DMSO) and diluted in *N*-2-hydroxyethylpiperazine-*N*-2-ethane sulfonic acid (HEPES) buffer (20 mM pH 7.4) to a final concentration of 100 µM. The preparation was vortexed, sonicated 2 min in a water bath, and incubated on a rocker for 24 h at 4 °C. The sample was again lyophilized and resuspended in a NH_4_HCO_3_ (50 mM pH 8.5) buffer. The oligomeric Aβ1–42 (AβO) was separated from monomers (AβM) on a Superdex 75 10/300 GL column coupled to a liquid chromatography system (ÄKTA pure, GE Healthcare) (Additional file 1: Fig. S1A). The column was equilibrated with NH_4_HCO_3_ (50 mM pH 8.5) and 500 µl sample was injected. The peptides were eluted at a flow rate of 0.5 ml/min, were AβOs pass through the column in the void volume (hence being > 70 kDa size) and AβMs pass through around half a column volume later (hence being of low molecular weight). The AβO and AβM were collected, lyophilized overnight and finally resuspended in PBS (Gibco). The peptides were quantified spectrophotometrically at 215 nm on a NanoVue (GE Healthcare) and stored at − 80 °C.

For Aβ fibrils, the AβO were incubated at 37 °C for 1–3 days for transmission electron microscopy** (**TEM) and dot blotting and 1–5 days for thioflavin T (ThT) assays.

### AβO characterization

To characterize the oligomer size, the AβO and AβM were run on a 4–20% SDS page gel (Expedeon) upon equal loading at 130 V (Additional file 1: Fig. S1B). AβO were also analyzed on a Nanosight ns300 (Malvern Panalytical) to track the particle size at a detection threshold of 6 and camera level 16. AβM were not analyzed due to the minimum particle size detection (10–40 nm) of the machine (Additional file 1: Fig. S1C). To characterize the oligomer structure, AβO were negative stained for TEM. The sample was prepared by letting 5 µl AβO (5 µM) be absorbed onto a Formvar carbon-coated 300 mesh copper grid, followed by two 30 s washes with distilled water and one wash with 2% uranyl acetate. Excess of uranyl acetate was removed using a lens paper and the grid was allowed to dry for 10 min. The sample was examined in a JEOL JEM-1230 electron microscope at 100 kV voltage (Additional file 1: Fig. S1D).

### ThT assay for analysis of Aβ fibril formation

To assess ThT fluorescence upon binding to Aβ fibrils formed in the absence and presence of TC-5RW at different time periods, we prepared a mixture of 5 μl Aβ fibrils in the absence and presence of TC-5RW (10 μg/ml) with 995 μl of 5 μM ThT solution (Sigma, USA) in glycine–sodium hydroxide buffer at pH 8.5. Each sample was thoroughly mixed and added to the transparent glass cuvettes. The cuvettes were inserted into a spectrofluorometer and the ThT fluorescence intensity was measured at the corresponding excitation emission (445 nm and 490 nm) wavelengths. We repeated each experiment at least three times and the values were recorded in triplicate, and ThT blank readings were subtracted from the corresponding values of each sample.

### TEM analysis of Aβ fibrils

We analyzed morphological characteristics of the samples with TEM (Hitachi H7650; Hitachi High-Technologies), with an acceleration voltage of 80 kV. In brief, 5 μl of the sample was placed on glow-discharged, formvar-coated grids and dip stained with 2% uranyl acetate and thoroughly air-dried before imaging.

### Dot blot analysis

Three μl of 10% brain homogenates, 2 μl of conditioned cell media and 3 μl of Aβ monomeric, oligomeric and fibril solution were spotted on nitrocellulose membranes (0.45 µm; Bio-Rad, USA) and completely air-dried at least for 15–30 min at room temperature. Membranes were incubated in 5% nonfat dry milk in 1× TBST for 1 h at room temperature. Membranes were incubated with Aβ (6E10), Aβ (B4) and Aβ (4G8) antibodies overnight and then processed as described for immunoblotting.

### Nondenaturing polyacrylamide gel electrophoresis (PAGE)

We performed immunoblotting under non-denaturing conditions according to a previously published method [60] and using the commercially available pre-cast Mini-PROTEAN TGX Gels 4–20% (Cat. # 4561093; Bio-Rad), Native Sample Buffer (Cat. # 1610738; Bio-Rad) and 10× Tris/Glycine Buffer (Cat. # 1610734; Bio-Rad) according to manufacturer’s instructions. Immunoblotting, primary and secondary antibodies incubation and development were done as described above for SDS-PAGE.

### Cell culture and treatments

Human neuroblastoma SH-SY5Y cells, murine microglial BV2 cells and the murine astrocyte C8D1A cells were cultured in Dulbecco’s Modified Eagle’s Medium (DMEM) and murine neuroblastoma N2a cells were cultured in Opti-MEM Glutamax medium (Gibco Life Technologies, Grand Island, NY, USA) with 10% fetal bovine serum (FBS), and penicillin/streptomycin at 37 °C in a 5% CO_2_ atmosphere. After the cells reached 70–80% confluence they were treated with 2.5–5 μM AβO or lipopolysaccharide (LPS, 1 μg/ml) with or without TC-5RW (10 µg/ml) and incubated for 24 h. After 24 h the cell lysates and conditioned media were processed for immunoblotting and dot blotting.

### Immunofluorescence and confocal microscopy of cells

SH-SY5Y, N2a, astrocytes (C8D1A) and BV2 cells were seeded (2 × 10^4^/ml) in chamber slides (ThermoFisher Scientific 75 Panorama Creek Drive Rochester, NY14625-2385, USA). After the cells reached 70–80% confluence they were treated with AβO 2.5–5 μM or LPS (1 μg/ml) with or without TC-5RW 10 μg/ml and incubated for 24 h. Then, cells were washed with 0.01 M PBS, fixed with 4% paraformaldehyde, and again washed with 0.01 M PBS twice for 5 min each, followed by permeabilization for 10 min using 0.1% Triton X-100 in 0.01 M PBS at room temperature. Following blocking for 1 h in blocking solution (2% serum and 0.1% Triton X-100 in 0.01 M PBS) cells were incubated with primary antibodies in blocking solution for overnight. After three washes (5 min each) in PBS, secondary antibody (Alexa Fluor^TM^ 555 goat anti-mouse secondary antibody, Invitrogen − 1:500) was added for 1 h at RT. Nuclei were stained with DAPI for 10 min and after final washes, coverslips were mounted using Mounting Medium (PermaFluor™, Thermo fisher). Images were collected and processed using a confocal laser scanning microscope (Zeiss LSM 700) and all the images were taken using the same conditions. The number of original confocal images obtained per well of the chamber slide was five per group and the images were converted into TIF images. The fluorescence intensity was measured using ImageJ software (National Institutes of Health, Bethesda, MD, USA). The immunofluorescence intensity was analyzed and expressed as the relative integrated density.

### MTT (3-[4,5-dimethylthiazol-2-yl]-2,5-diphenyltetrazolium bromide) assay for cell viability

MTT assay was performed according to the manufacture protocol (Roche, Germany) and our previously published procedure [57]. Briefly, N2a cells were cultured in 96-well plates (1 × 10^5^ cells/well). After 72 h of incubation and 70–80% confluency the N2a cells were treated with 10 μg/ml and 25 µg/ml TC-5RW, or 5 μM AβO with or without 10 μg/ml TC-5RW for 24 h. MTT (5 mg/ml in PBS) solution was added and the plates were incubated for 4 h at 37 °C. DMSO was added to the wells, and the plates were agitated for 10–20 min to dissolve formazan crystals. The absorbance was measured at 550–570 nm (*L*1) and 620–650 nm (*L*2) using a scanning microplate reader. The L2 absorbance measures cell debris and well imperfections. The absorbance (*A* = *L*1 − *L*2) of each well was used to calculate the percentage of cell survival as × 100 absorbance of treated wells/absorbance of control wells.

### Data representation and statistical analyses

The scanned immunoblot images and confocal tiff images were analyzed using Image J software. The quantification of immunostaining results was produced from the raw integrated density of immunofluorescence intensity for immunoreactivity of each staining assay in image J. The raw integrated density values were converted to single-digit relative integrated density values by dividing them by their average values. Similarly, for immunoblotting we did the same calculation and analysis. For immunoblotting we analyzed the raw band density for each marker and beta actin and then correspondingly divided by their average numbers, which gave us the relative integrated density values. For statistical analysis we used two-tailed independent Student’s *t*-test for two groups or for multiple groups, one-way/two-way analysis of variance (ANOVA) followed by Tukey’s post hoc, as applicable. The histograms were produced using GraphPad Prism software (GraphPad 8, Software, USA). Significance = *p ≤ 0.05, **p ≤ 0.01, ***p ≤ 0.001 and ****p ≤ 0.0001.

## Results

### TC-5RW inhibits Aβ aggregation and reduce Aβ toxicity and immunoreactivity in in vitro

**Fig. 1:**
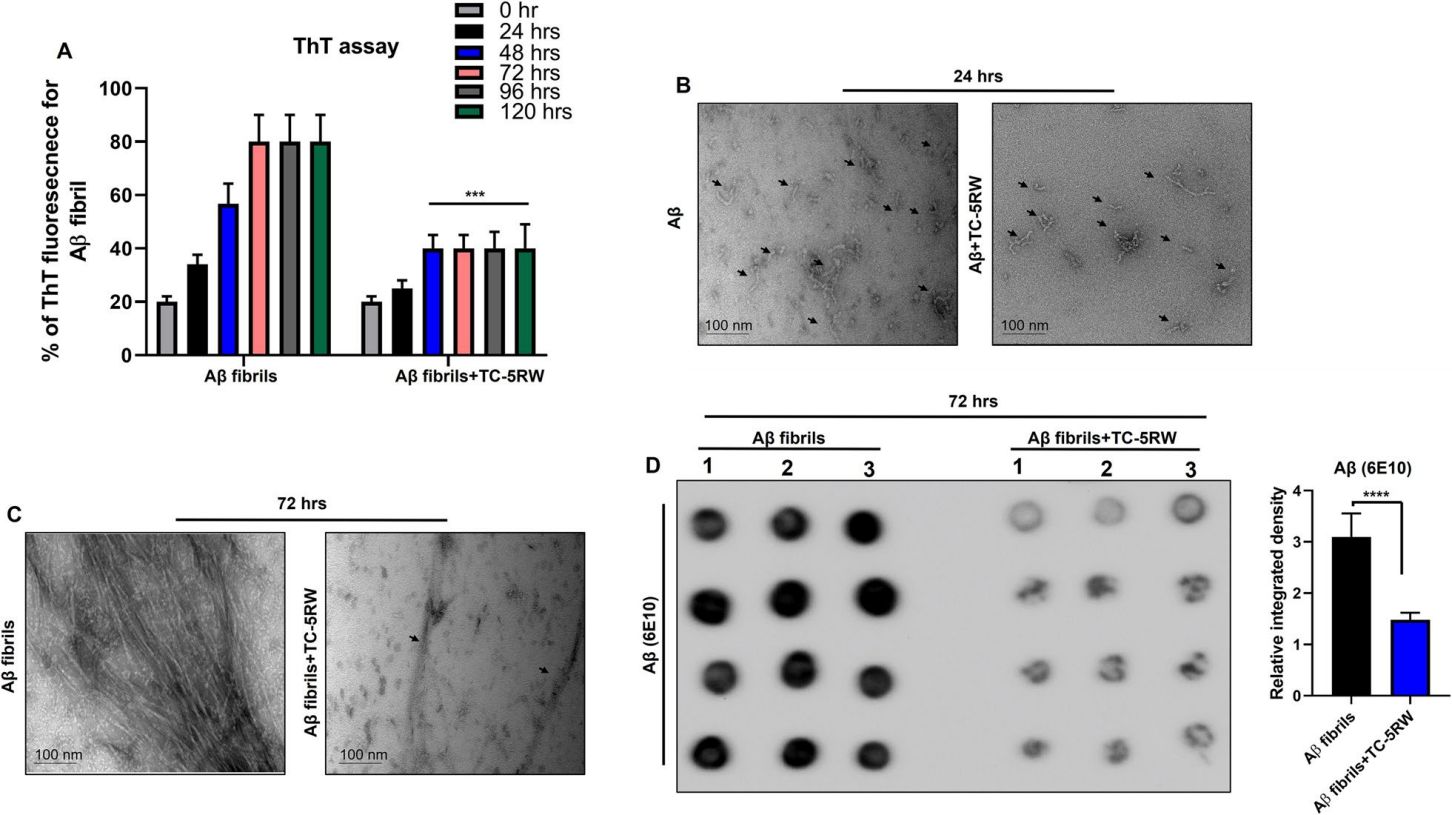
TC-5RW inhibits Aβ aggregation in vitro. **A** Histogram represents the % of ThT fluorescence, revealing that Aβ aggregation is reduced by TC-5RW. Significance = ***p < 0.001. **B** TEM images of Aβ fibrils after incubation at 37 °C for 24 h with and without TC-5RW (10 µg/ml). **C** TEM images of Aβ fibrils after incubation at 37 °C for 72 h with and without TC-5RW (10 µg/ml). **D** Dot blotting of Aβ fibrils (using mAb 6E10) after incubation at 37°C for 72 h with and without TC-5RW (10 µg/ml). Dots in lanes 1, 2, and 3 represent quadruplet from three different tubes for each group. Histogram represents the means ± SEM from three independent experiments. Significance = **p < 0.0001

To examine whether TC-5RW has an inhibitory effect on Aβ aggregation and fibril formation, we performed ThT fluorescence assay. We incubated AβO with or without TC-5RW (10 µg/ml) at 37 °C for 5 days. We performed ThT assay at the start of day 1 which is set as 0 h and then every 24 h until 120 h for all samples. We observed a time-dependent gradual increase in the percentage (80%) of ThT fluorescence intensity indicative of Aβ fibril formation while TC-5RW (10 µg/ml) significantly reduced ThT fluorescence intensity (40–45%) and kept it constant after 24 h (Fig. 1A; ***p < 0.001). We also performed TEM to validate the ThT results by visualizing Aβ fibrils after 24 and 72 h at 37 °C in the absence and presence of TC-5W (10 µg/ml). The TEM results showed small protofibrils after 24 h and fibrils after 72 h incubation. Interestingly, we observed a reduction of protofibrils and in particular, large fibrils in the presence of TC-5RW (10 µg/ml), respectively, at 24 h and 72 h (Fig. 1B, C). Overall, both ThT assay and TEM results indicate that TC-5RW has prevented Aβ fibril formation.

Further, to examine the effect of TC-5RW (10 µg/ml) on Aβ fibrils, we incubated preformed Aβ fibrils in the absence and presence of TC-5RW (10 µg/ml) with the same conditions used for TEM analysis for 72 h followed by dot blot analysis, using a membrane with 0.45 µm pore size that most efficiently binds proteins with a molecular weight of > 20 kDa. The dot blot results showed significantly lower immunoreactivity of Aβ (6E10) antibody for the co-incubation of Aβ fibrils with TC-5RW (10 µg/ml) as compared to control Aβ fibrils (Fig. 1D). Further to assess the effect of TC-5RW on high molecular weight fibrils and lower molecular weight Aβ oligomeric/monomeric species, we performed immunoblotting under non-denaturing conditions according to a previously published protocol [60]. The immunoblotting results indicated that at 37 °C for 72 h less Aβ fibrils formed in the presence of TC-5RW (10 µg/ml) as compared to the Aβ fibrils formed in the absence of TC-5RW (Additional file 1: Fig. S2; ***p < 0.001). Most importantly, under these non-denaturing conditions we were not able to detect oligomeric/monomeric species of Aβ (Additional file 1: Fig. S2).

To assess whether TC-5RW has detrimental effects on cells, we performed MTT assay using mouse neuroblastoma N2a cells. We found that both doses of TC-5RW (10 µg/ml and 25 µg/ml) have no adverse effect on neuronal cells (Additional file 1: Fig. S3). Next, we aimed to determine whether Aβ fibrils formed in the presence of TC-5RW (10 µg/ml and 25 µg/ml) are less neurotoxic as compared to the Aβ fibrils formed in the absence of TC-5RW (10 µg/ml and 25 µg/ml). MTT results showed that Aβ fibrils in presence of both doses of TC-5RW significantly reduced Aβ toxicity and increased the cell viability as compared to Aβ fibrils formed in the absence of TC-5RW (Additional file 1: Fig. S3; *p < 0.05).

**Fig. 2:**
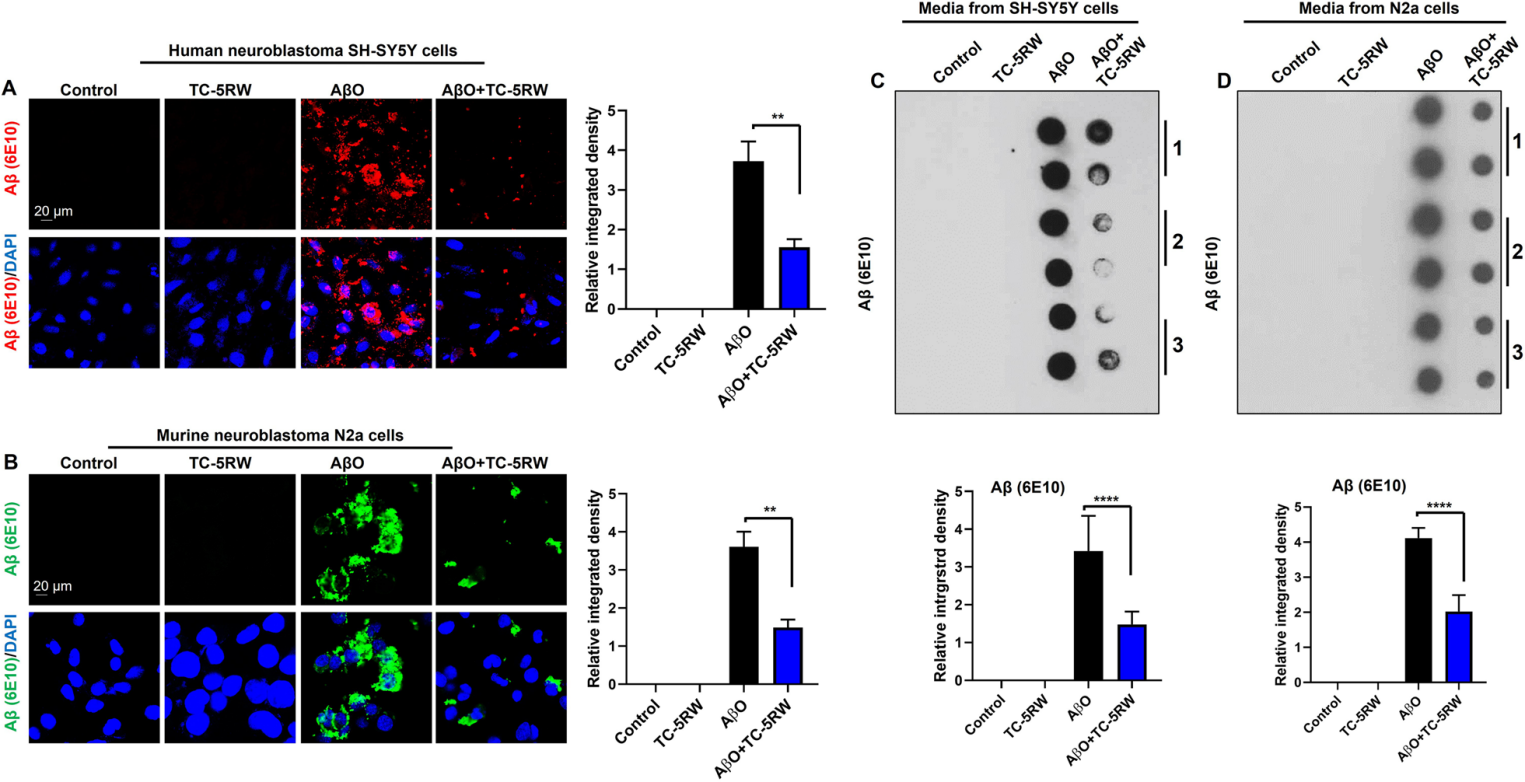
TC-5RW reduces intracellular Aβ immunoreactivity and extracellular levels of Aβ in vitro. **A** Human neuroblastoma SH-SY5Y cells were incubated with amyloid beta oligomer (AβO) (5 µM) in the presence or absence of TC-5RW (10 µg/ml). Aβ (6E10) (red) and DAPI (blue). **B** Murine neuroblastoma N2a cells were incubated with AβO (5 µM) in the presence or absence of TC-5RW (10 µg/ml). Aβ (6E10) (green) and DAPI (blue). For both (**A**, **B**), magnification: 63X. Scale bar = 20 μm. Histogram represents the means ± SEM for the representative groups (*n* = 3) and the number of independent confocal microscopy experiments = 3. Significance = **p < 0.01. **C**, **D** Dot blotting and quantification of immunoreactivity of Aβ (6E10) in conditioned media of SH-SY5Y and N2a cells that were incubated with AβO (2.5–5 µM) in the presence or absence of TC-5RW (10 µg/ml). Dots in each groups represent duplicates from the conditioned media of three independent in vitro experiments. Histograms represent as the means ± SEM for the representative media of three independent experiments

Next, we treated the human neuroblastoma SH-SY5Y cells and N2a cells with AβO (2.5–5 µM) and TC-5RW (10 µg/ml) and performed immunocytochemical staining using Aβ (6E10). The confocal results showed that TC-5RW treatment significantly reduces the immunoreactivity of Aβ detected with Aβ (6E10) as compared to controls without TC-5RW in both SH-SY5Y cells and N2a cells (Fig. 2A, B).

Additionally, using dot blot analysis we measured the amount of Aβ in the conditioned media of SHSY-5Y and N2a cells incubated with AβO (2.5–5 µM) in the presence or absence of TC-5RW (10 µg/ml) for 24 h. We found that TC-5RW significantly reduced the immunoreactivity of Aβ in the media using Aβ6E10 (Fig. 2C, D) and Aβ (4G8) and Aβ (B4) antibodies (Additional file 1: Fig. S4A–D). These results suggest that TC-5RW prevented Aβ content and reduced Aβ-induced neurotoxicity in neuronal cells. Further studies will need to clarify whether TC-5RW enhances degradation of AβO, or forms complexes with AβO that result in reduced toxicity and potentially alterations in immunoreactivity.

### Effect of TC-5RW treatment on Aβ levels and Aβ plaques in 5XFAD mouse model

**Fig. 3:**
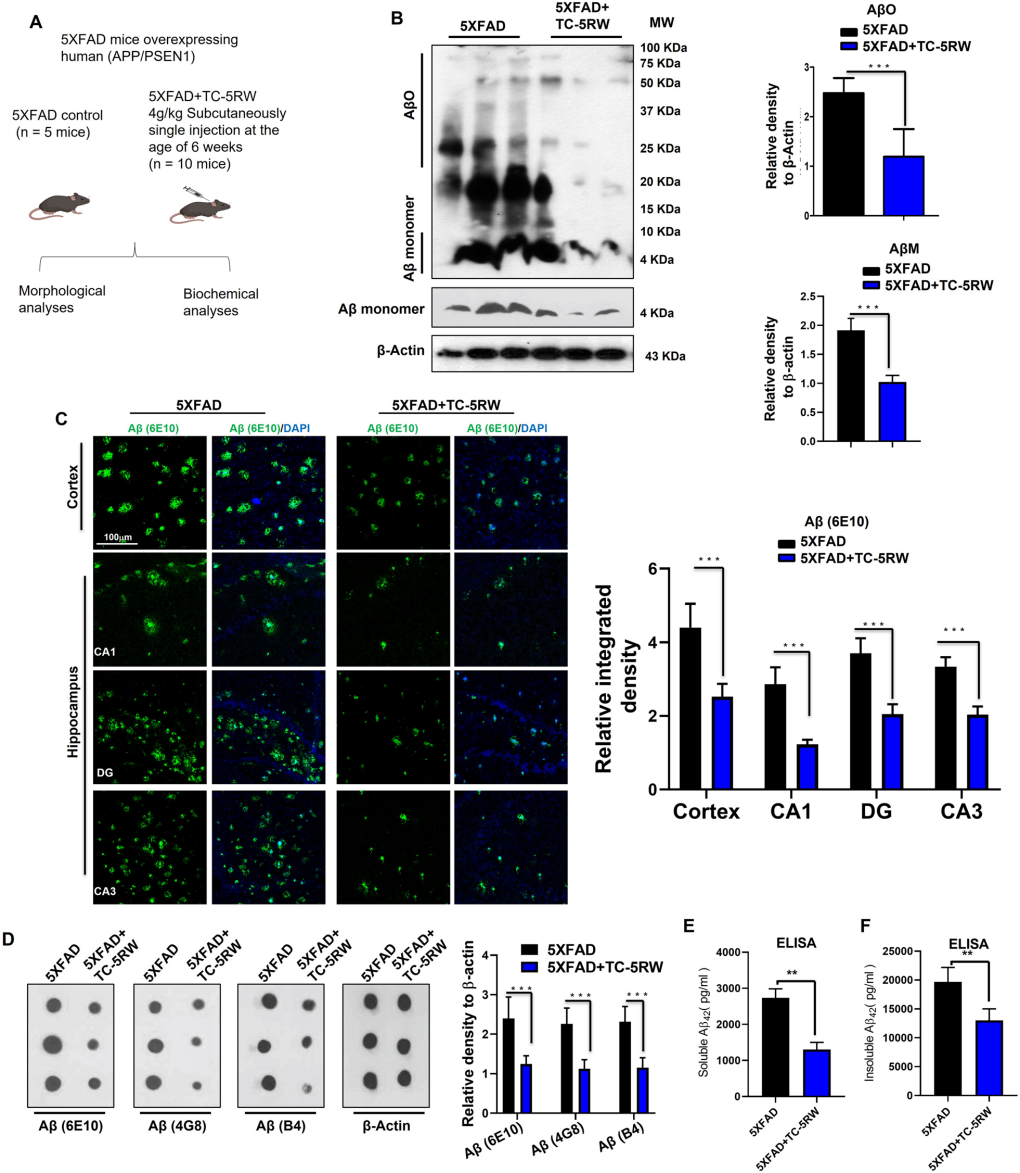
Treatment of TC-5RW significantly reduce AβO levels, Aβ plaques and its associated pathologies. **A** Schematic representation of grouping and treatment of mice for cohort 1 experiment. **B** Immunoblotting and quantification of AβO and AβM in the brains of 5XFAD mice treated or not with a single dose of TC-5RW. β-actin served as a loading control and was obtained after stripping of the same membrane of combined AβO. For obtaining the amyloid monomer with clear separation, we performed immunoblotting using 16% tris–tricine gel with 2.5 µm PVDF membrane. Histograms represent the means ± SEM for the representative proteins (*n* = 3 mice/group) and the number of independent immunoblotting experiments = 3. Uncropped immunoblotting images shown in Additional file 1: Fig. S8A, B. We used the representative cropped AβM in Additional file 1: Fig. SB for quantification. **C** Confocal microscopy images of Aβ (6E10) (green) and DAPI (blue) in the cortices and hippocampi of 5XFAD mice treated or not with TC-5RW. Histogram represents as the means ± SEM for *n* = 3 mice/group, and the number of independent confocal microscopy experiments = 3. Magnified 10×. Scale bar = 100 μm. **D** Dot blotting and quantification of Aβ (6E10), Aβ (4G8) and Aβ (B4) and in the brain homogenates of non-treated 5XFAD and TC-5RW-treated 5XFAD mice. β-actin served as a loading control. Histogram represents as the means ± SEM for the representative proteins (*n* = 3 mice/group) and the number of independent dot blotting experiments = 3. Uncropped dot blotting images shown in Additional file 1: Fig. S8C. **E**, **F** Analyses of relative soluble Aβ42 and relative insoluble Aβ42 levels in the brain hemisphere homogenates of the 5XFAD mice treated or not with TC-5RW using commercially available ELISA. Histograms represent as the means ± SEM for the indicated proteins (*n* = 5 mice/group). Significance = *p < 0.05; **p < 0.01; ***p < 0.001. Panel **A** image was prepared using Biorender.com

To investigate the beneficial and therapeutic effect of TC-5RW on Aβ pathologies, we chose to use 5XFAD mice, a mouse model of familial AD characterized by rapid Aβ generation and aggregation in the brain [61]. We used two groups of 5XFAD mice. One group was treated with only a single dose of TC-5RW (4 g/kg/SC) at the age of 6 weeks, while the other group was left untreated and served control. Mice of both groups were euthanized at 10 months and brains were collected for biochemical and immunohistochemical analyses (Fig. 3A). We performed immunoblotting using the Aβ6E10 antibody. The immunoblotting results indicated that TC-5RW reduced the AβM and AβO levels as compared to non-treated 5XFAD mice. We quantified AβO and AβM level, which showed that TC-5RW significantly reduced AβO levels as compared to non-treated 5XFAD mice (Fig. 3B; ***p < 0.001). 5XFAD mice present with extracellular Aβ plaques, which are a primary hallmark of AD pathologies [61]. Thus, to examine the effect of TC-5RW on Aβ plaques we performed immunofluorescence staining using Aβ6E10 antibody. Results showed that TC-5RW significantly reduced the Aβ plaque burden and Aβ immunoreactivity in the cortical and hippocampal regions of 5XFAD mouse brains as compared to brains from non-treated 5XFAD mice (Fig. 3C; ***p < 0.001). Dot blot analysis of brain homogenates and its quantification confirmed that TC-5RW remarkably reduced the Aβ (6E10), Aβ (4G8) and Aβ (B4) immunoreactivity as compared to non-treated 5XFAD mice (Fig. 3D). Next, we used 10% brain homogenates of both groups of mice to measure the overall content of soluble and insoluble Aβ42 by ELISA. The ELISA results were very consistent and showed significantly reduced concentrations (pg/ml) of both soluble and insoluble Aβ42 in TC-5RW treated mice as compared to non-treated 5XFAD mice (Fig. 3E, F; **p < 0.01). In summary, these in vivo results recapitulate the in vitro results and suggest that TC-5RW treatment of 5XFAD mice significantly reduces AβM, AβO and plaque load in the brains as well as the soluble and insoluble form of Aβ42.

### TC-5RW attenuates proinflammatory marker GMFβ and glial activation in vivo and in vitro

**Fig. 4:**
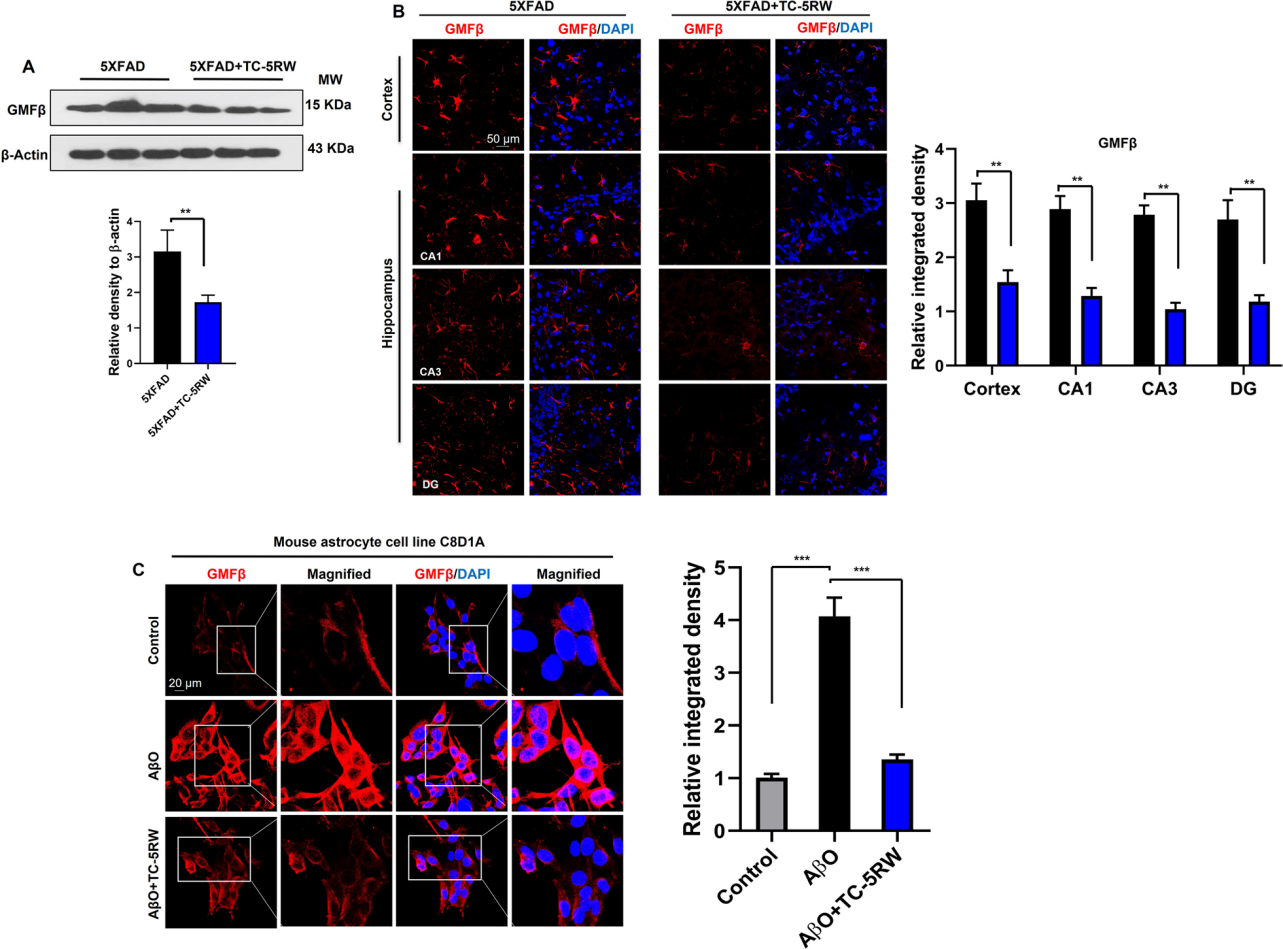
TC-5RW reduces activated GMFβ in the brains of 5XFAD mice. **A** Immunoblotting and quantification of proinflammatory GMFβ in the brains of untreated and treated 5XFAD mice. β-actin served as a loading control. Histogram represents the means ± SEM for the representative proteins (*n* = 3 mice/group) and the number of independent immunoblotting experiments = 3. Uncropped immunoblotting images shown in Additional file 1: Fig. S9. **B** Confocal images of GMFβ (red) (DAPI, blue) staining in the cortices and hippocampus regions (CA1, CA3 and DG) in brains of untreated and treated 5XFAD mice. Histogram represents the means ± SEM for *n* = 3 mice/group, and the number of independent confocal microscopy experiments = 3. Magnification: 63×. Scale bar = 50 μm. Magnification: 63×. **C** Confocal images of GMFβ (Red) (DAPI, Blue) of C8D1A astrocytic cells incubated with AβO (5 µM) and treated or untreated with TC-5RW (10 μg/ml). Magnification: 63×. Scale bar = 20 μm. Histogram represents as the means ± SEM for *n* = 3 /group, and the number of independent in vitro and confocal microscopy experiments = 3. Significance = *p < 0.05; **p < 0.01; ***p < 0.001

**Fig. 5:**
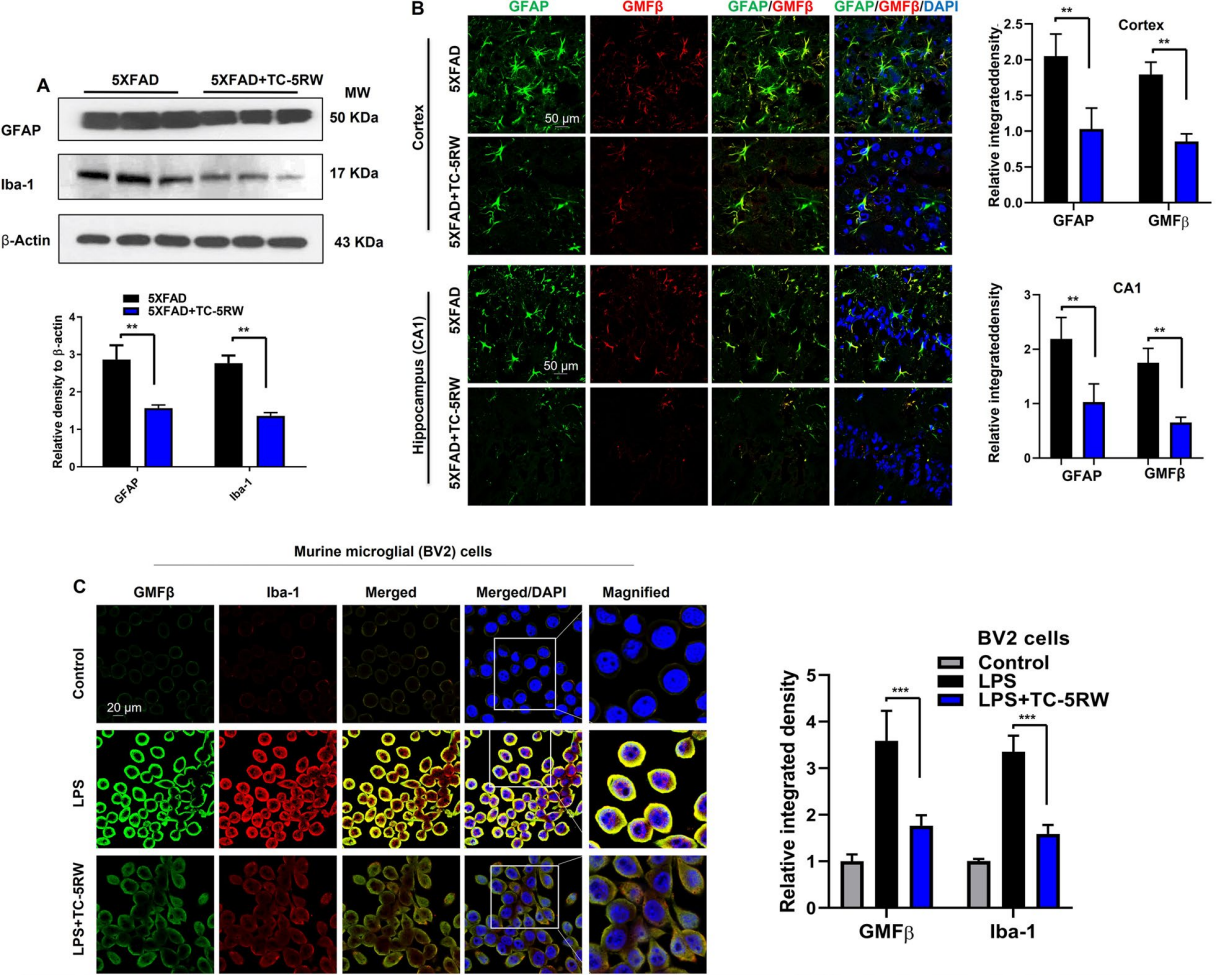
TC-5RW reduces the activated gliosis in the brain of 5XFAD mice. **A** Immunoblotting and quantification of GFAP, Iba-1 and β-Actin in the brain homogenates of non-treated 5XFAD and TC-5RW-treated 5XFAD mice. β-Actin was used as a loading control. Histograms represent the means ± SEM for the representative proteins (*n* = 3 mice/group) and the number of independent immunoblotting experiments = 3. Significance = *p < 0.05. Uncropped immunoblotting images shown in Additional file 1: Fig. S10A, B. **B** Confocal images of GFAP (green) and GMFβ (red) in the cortex and hippocampus (CA1) region in brains of non-treated and TC-5RW-treated 5XFAD mice. Histogram represents the means ± SEM for *n* = 3 mice /group, and the number of independent confocal microscopy experiments = 3. Magnification: 63×. Scale bar = 50 μm. Significance = ***p < 0.001. **C** Double immunocytochemistry results of GMFβ (green) and Iba-1 (red) in microglial BV2 cells, which were incubated with LPS (1 µg/ml) in the absence and presence of TC-5RW (10 µg/ml) for 24 h. Magnification: 63×. Scale bar = 20 μm. Histogram represents as the means ± SEM for *n* = 3/group, and the number of independent in vitro and confocal microscopy experiments = 3. Significance = ***p < 0.001

It is well established that inflammation significantly contributes to the onset and progression of neuroinflammatory and neurodegenerative diseases including AD [41, 62–64]. With regard to proinflammatory mediators, GMFβ is an emerging marker in AD pathologies. Several studies reported the upregulation of GMFβ expression in glial cells associated with misfolded Aβ and tau protein in the cortex and hippocampus of AD brains [40, 65]. Teruya et al. reported that CE’s effective response is affected by GMFβ and its proximity genes [36]. Therefore, we assessed the effect of TC-5RW on the level of GMFβ in in vivo and in vitro AD models. We examined GMFβ by immunoblot and confocal microscopy analysis of 5XFAD mouse brains. The immunoblotting results revealed that TC-5RW treatment significantly ameliorated elevated level of GMFβ in 5XFAD mice as compared to non-treated 5XFAD mice (Fig. 4A; **p < 0.01). These findings were supported by confocal microscopy results, which demonstrated that TC-5RW significantly reduced the GMFβ immunoreactivity in both cortical and hippocampal (CA1, CA3 and DG) tissues of 5XFAD mice as compared to non-treated 5XFAD mice (Fig. 4B; **p < 0.01). Of note, Ahmed et al. also found an elevated level of GMFβ in activated glial cells in the cortical and hippocampal region of the brain in early AD (5XFAD) mouse model, which supported the hypothesis that increased levels of AβO species trigger the activation of the proinflammatory marker GMFβ. Thus, we designed in vitro experiment where astrocytic C8D1A cells were treated with AβO in the absence and presence of TC-5RW (10 µg/ml). After 24-h incubation, the cells were processed for immunocytochemical staining of GMFβ and confocal microscopy was performed. The results and quantifications shown in Fig. 4C indicated that TC-5RW (10 µg/ml) treatment significantly attenuates GMFβ immunoreactivity as compared to AβO-only treated cells (***p < 0.001). Further, we performed immunoblot analysis of 5XFAD brain homogenates using GFAP, a marker for activated astrocytes, and Iba-1, a marker for activated microglia, respectively, to determine glial activation with and without TC-5RW treatment. We found that TC-5RW treatment significantly reduced the elevated level of GFAP and Iba-1 in 5XFAD mice as compared to non-treated 5XFAD mice (Fig. 5A; **p < 0.01). We also performed confocal microscopy to affirm morphologically the activation of astrocytes and microglia using GFAP and Iba-1, respectively, revealing that TC-5RW reduces the immunoreactivity of GFAP and Iba-1 in both cortical and hippocampal (CA1, CA3 and DG) as compared to non-treated 5XFAD mice (Additional file 1: Fig. S5; **p < 0.01). Next, we performed double-immunofluorescence to analyze GMFβ levels in the activated astrocytes. The double-immunofluorescence results showed that TC-5RW treatment significantly reduced the immunoreactivity of GMFβ and GFAP in both cortical and hippocampal (CA1, CA3 and DG) as compared to non-treated 5XFAD mice (Fig. 5B; **p < 0.01; Additional file 1: Fig. S6; **p < 0.01).

In order to determine whether the reduction of glial activation and neuroinflammatory markers are a consequence of reduced Aβ accumulation or directly affected by TC-5RW, we tested the anti-inflammatory effect of TC-5RW on LPS-mediated glial activation. The murine microglial cell line BV2 was incubated with LPS (1 µg/ml) in the absence or presence of TC-5RW (10 µg/ml) for 24 h and performed confocal microscopy, which showed that TC-5RW reduced immunoreactivity of GMFβ and Iba-1 in LPS activated BV2 cells (Fig. 5C; **p < 0.01). Next, the murine astrocytic C8D1A cells were incubated with LPS (1 µg/ml) in the absence or presence of TC-5RW (10 µg/ml) for 24 h and performed confocal microscopy for double-immunofluorescence for GFAP and GMFβ in astrocyte cell line C8D1A. The double-immunofluorescence results indicated that TC-5RW reduced immunoreactivity of GMFβ and GFAP in LPS activated C8D1A cells (Additional file 1: Fig. S7). Taken together, these results suggest that TC-5RW can prevent neuroinflammation independent of Aβ pathologies as well as neuroinflammation associated with Aβ in familial AD.

### Single and chronic administration of TC-5RW treatment significantly improve cognitive functions of 5XFAD mice

**Fig. 6:**
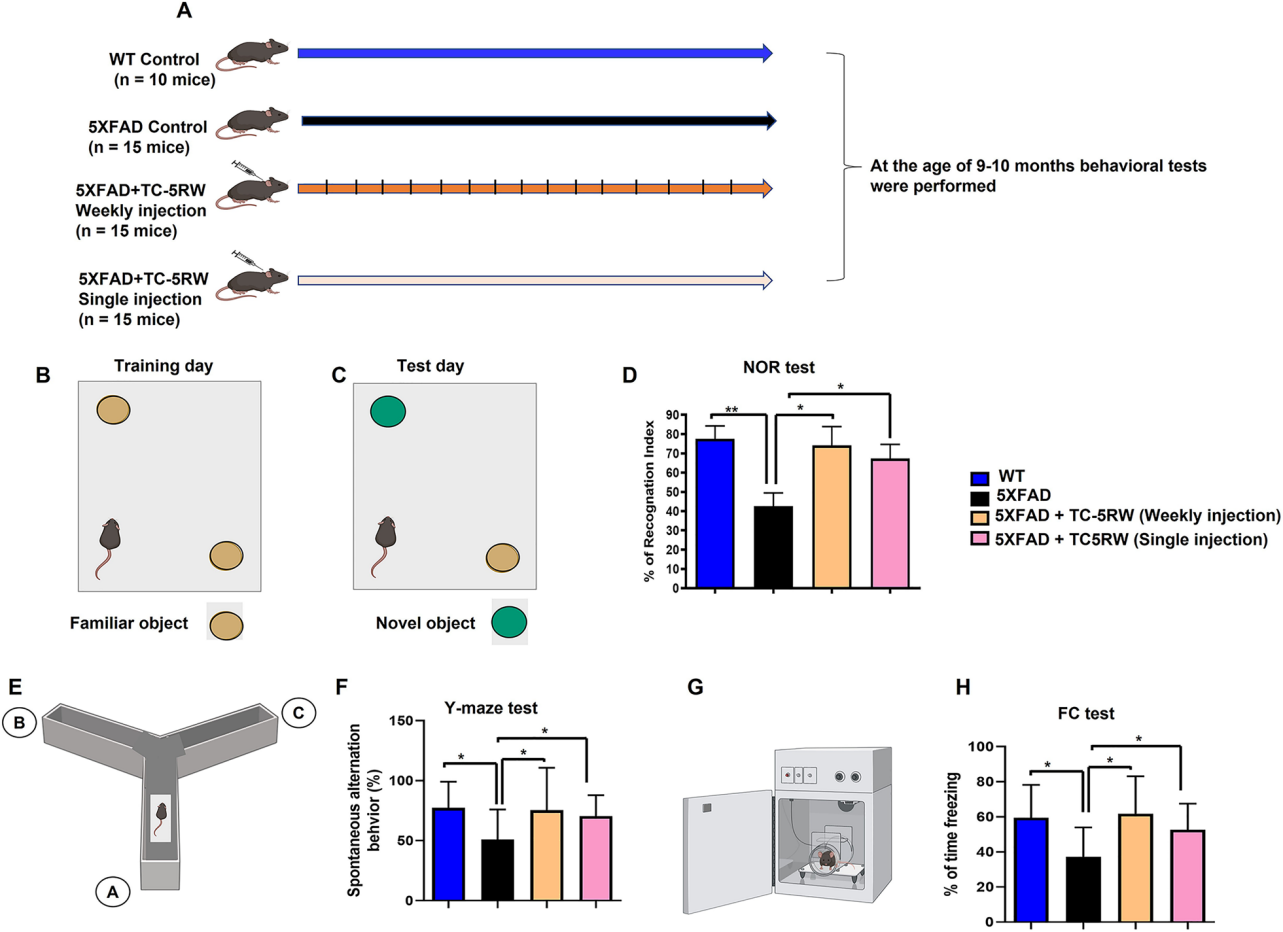
Single and chronic administration of TC-5RW treatment significantly improve cognitive functions of 5XFAD mice. **A** Schematic representation of grouping and treatment of mice for cohort 2 experiment. To third and fourth groups TC-5RW was infused very slowly and carefully subcutaneously (SC) at the neck of mice. **B**, **C** Schematic representation of training and test phase for the NOR test. **D** Histogram represents the % index in testing session of NOR test. **E** Schematic representations of Y-maze with three arms **A**, **B** and **C**. **F** Histogram represents the spontaneous alteration behavior percentage of mice during Y-maze test. **G** Schematic representations of chamber used for FC test. **H** Histograms represent the % of freezing during the FC test. The histograms data represent the average for the mice (WT = 10 male mice/group, all other groups 15 male mice/group). Significance = *p < 0.05, **p < 0.01. WI: Weekly injection of TC-5RW to 5XFAD mice; SI: single injection of TC-5RW to 5XFAD mice. Most of the images of panels **A**–**C**, **E** and **G** were prepared using Biorender.com

To further validate our biochemical and immunohistochemical results and to assess for the first time the memory improving effect of TC-5RW in 5XFAD mice, we designed another study using WT mice and three groups of 5XFAD mice, including groups with single or weekly treatment with TC-5RW (Fig. 6A).

We initially performed the NOR test to evaluate the learning and recognition memory functions of rodents [66–69]. This task is based on the natural tendency of rodents to investigate novelty. A mouse is allowed to explore two identical objects kept in a context, and after an inter-trial interval is re-exposed to the context having one familiar object, and a novel object. Mice having normal memory functions investigate the novel object preferentially. In NOR test, on the test day we observed that non-treated 5XFAD mice spent less time for exploration of novel object and % of relative recognition indices as compared to WT mice. Most importantly, we found a time increase of exploration for novel object and % of recognition index for both single and chronic treatment with TC-5RW of 5XFAD mice as compared to non-treated mice (Fig. 6B–D; *p < 0.05).

Following NOR test, we performed Y-maze test to assess the short-term spatial learning and memory function of mice. The Y-maze test results demonstrated that WT mice have the ability to visit each arm but also have the tendency to not visit the same arm repeatedly. Similarly, the 5XFAD mice treated with single and chronic treatment of TC-5RW showed increased alternation behavior to visit each arm and avoid the previously visited arm. Overall, WT and 5XFAD mice treated with TC-5RW showed significantly higher percentage of alternation as compared to non-treated 5XFAD mice (Fig. 6E, F; *p < 0.05).

Next, in order to examine the effect of TC-5RW treatment on consolidated learning and memory function, we performed FC test. The results of FC test showed that non-treated 5XFAD mice have less % of freezing and total time of freezing as compared with WT mice, while both single and chronic treatment with TC-5RW resulted in a significant increase in % of freezing and total time of freezing as compared to non-treated 5XFAD mice (Fig. 6G, H; *p < 0.05).

In conclusion, our intriguing findings indicate that TC-5RW induced beneficial and therapeutic effects via inhibiting Aβ aggregation and neuroinflammation, as well as improved learning and memory functions in transgenic 5XFAD mouse model of AD.

## Discussion

Protein misfolding neurodegenerative diseases are devastating and incurable brain disorders, including AD. AD poses a huge socio-economic burden on the health care system with the prospect of case numbers tripling over the next 30 years. Hence, treatments are needed to address the growing prevalence of AD. However, drug development for AD and related neurodegenerative diseases has proven to be a very challenging and onerous task for researchers, clinicians, and pharmaceutical industries. Development of new therapeutics is extremely costly and time consuming and leaves uncertainty about their safety. Alternatively, testing repurposed, FDA-approved drugs is an attractive avenue of research because these drugs are relatively economical, safe and accessible as well as have a high potential to expedite translation into clinical trials [70, 71]. It is worthwhile to use innovative and rationale approaches to investigate FDA-approved compounds for repurposing to expedite the development of novel and safe therapeutics to treat AD. Recently, we and other groups tested FDA-approved CEs in prion diseases and found that CEs have therapeutic effects by inhibiting propagation of prions, infectious agents generated upon misfolding of the host’s cellular prion protein and prototypic for other misfolded proteins associated with neurodegenerative diseases, and extend the survival of prion-infected mice [30–35]. Herein, we tested and validated for the first time FDA-approved CE (HPMC) representative TC-5RW (Type E (Hypromellose 2910 classified by the United States Pharmacopeia)) as a potential, emerging and effective compound to halt and treat familial AD. We demonstrated that both single and chronic treatment with TC-5RW significantly enhanced cognitive functions of 5XFAD mice. TC-5RW has the ability to prevent Aβ aggregation and accumulation both in in vitro and in vivo models. TC-5RW acts as a potent protective agent to rescue Aβ-mediated neuroinflammation via regulating proinflammatory mediators and glial activation.

Numerous research findings and literature reviews reported that the amyloidogenic pathway is a primary and the most significant pathological signature in AD pathologies. The oligomerization of Aβ monomers is a vital step of AβO-induced neurotoxicity and amyloid fibril formation which subsequently produce insoluble deposits of Aβ plaques [72, 73]. The accumulation of Aβ aggregates has been shown in human post-mortem brain and various transgenic mouse models of AD, including the 5XFAD mouse model of familial AD. The presence of Aβ fibrils and AβO is neurotoxic and triggers neuroinflammation, which subsequently leads to neurodegeneration and memory dysfunction [42, 74]. Numerous studies reported that soluble AβO species spread among cells and neuropil and therefore, were considered as a main mediator of synaptic and apoptotic neurodegeneration, which subsequently lead to memory impairment in AD. Therefore, preventing and inhibiting formation of neurotoxic Aβ aggregates is one of the primary concerns to be targeted in early AD.

Several natural and synthetic compounds were tested to modulate the amyloidogenic pathway and aggregation. Numerous compounds have the potential to interfere with Aβ aggregation via restricting the conversion of Aβ monomers into AβOs, inhibiting amyloid fibrils formation and disrupting amyloid fibrils, as well as by activation of the non-amyloidogenic pathway and stimulation of other neuroprotective signalling pathways that rescue Aβ pathologies [75–77]. Notably, in the last two decades several clinical trials were conducted with different therapeutic approaches especially with active and passive immunotherapy to target the Aβ, unfortunately most have failed their endpoints. Importantly, the recent results of the phase-3 clinical trial of immunotherapy using lecanemab (a humanized IgG1 monoclonal antibody that binds with high affinity to Aβ soluble protofibrils) showed the reduction of Aβ burden in early AD patients and to some extent improved the cognitive functions of AD patients; however, lecanemab administration produced some adverse effects in a few patients [78]. Despite this, the evidence of clinical studies suggest that anti-amyloid therapy may not be the most effective approach to treat AD [75]. Several other factors contribute to the AD pathologies, in which inflammation has a key role and connects other pathological mediators with the main hallmarks of AD. Therefore, it is important to not only focus on the Aβ aspect of AD pathologies, but we have to consider multiple aspects, mainly inflammation to halt and treat AD. One strategy for combination therapy is to target the Aβ and other factors but the combination therapy may induce some detrimental side effects. Therefore, another possible approach is to identify effective and safe medication that not only inhibits the Aβ, but also could rescue other pathological mediators and improve memory functions. Our efforts are in line with this approach, using CE-derived FDA-approved TC-5RW to target Aβ aggregation and its mediated cascades which together cause learning and memory deficit. We confirmed in in vitro studies that TC-5RW prevents Aβ aggregation and Aβ-induced neurotoxicity. These results were further supported by several studies, which reported that macromolecules and polymers have the potential to halt misfolded protein aggregation. Our group recently showed that TC-5RW inhibit the propagation of prions, an infectious agent consisting of misfolded protein. TC-5RW was also beneficial in animal models of prion diseases, which is consistent with our findings that TC-5RW significantly reduces AβO levels, Aβ plaques and insoluble and soluble Aβ42 contents in the brains of 5XFAD mouse model of AD.

Mounting studies reported the significant role of neuroinflammation and the innate immune system in protein misfolding neurodegenerative diseases [79, 80]. At physiological conditions brain glial cells such as astrocytes play a key role in the development and function of neuronal cells. Neuroinflammation is triggered by glial cells in the central nervous system in response to the accumulation of misfolded proteins, infection, toxicity, or autoimmunity. It was shown that intracerebral administration of human Aβ1-42 in mouse brains caused glial activation, enhanced levels of proinflammatory GMFβ, and triggered the activation of various inflammatory cytokines and chemokines, which have roles in neuroinflammation [81]. Brain-specific GMFβ overexpression in astrocytes is responsible for the stimulation of granulocyte–macrophage colony-stimulating factor. It was further confirmed that secreted GMFβ in astrocytic conditioned media further instigate the production and secretion of other proinflammatory mediators such as tumor necrosis factor alpha, interleukin-1 beta, and interleukin-6 in microglia [38, 39]. It was shown that neuronal SH-SY5Y cells exposed to Aβ1-42 and GMFβ trigger mitochondrial apoptotic neurodegeneration via elevated Bax/Bcl2 expression and release of cytochrome-c, which subsequently leads to apoptosis. These studies suggest that GMFβ synergistically increases the detrimental effects of Aβ on the cellular homeostasis of neurons. This evidence indicated that proinflammatory GMFβ and glial activation in the presence of Aβ exacerbate AD pathologies [43]. Of note, in addition to the inhibitory effect of TC-5RW on Aβ aggregation, we found that TC-5RW acted as anti-inflammatory agent and reduced the elevated level of proinflammatory GMFβ and glial activation in in vitro and in vivo studies. Most importantly, both the single and chronic dosage regimen for TC-5RW treatment significantly improved the learning and memory functions of 5XFAD mice (Fig. 6A–C). Along with improving the memory functions of mice, our findings show that TC-5RW not only inhibits Aβ aggregation and reduces Aβ burden, but also acts as a potent anti-inflammatory and rescues neuroinflammation. Therefore, it is suggested that CEs or its derived compounds would be advantageous to those drugs which only have the ability to inhibit Aβ aggregation or neuroinflammation.

## Conclusions

In summary, our in vitro and in vivo results showed that TC-5RW inhibits AβO and Aβ fibril formation as well as prevents neuroinflammation and improves cognitive functions in a mouse model of familial AD. Nevertheless, besides these intriguing beneficial and therapeutic effects of CEs, we further recommend future studies to answer the following points and questions raised from our findings: (1) What is the exact underlying mechanism of CEs in inhibition of misfolded proteins and particularly inhibition of Aβ aggregation? Do CEs act like other macromolecules and their hydrophobic region binds to Aβ [82] and consequently prevent the fibrillation of Aβ and its neurotoxicity? Or do CEs inhibit the seeding of AβO in the same manner as inhibiting the propagation of prions [33, 35]. (2) It was reported that in prion disease the CEs’ effect was modulated via immune cells as well as the CEs therapeutic efficiency was also affected by the strain of mouse models in prion diseases [31, 36, 37], hence it will be important to decipher the role of CEs in the periphery and their effect in the brain. (3) Is there a beneficial and therapeutic effect of CEs in models of sporadic AD, particularly in ApoE4-related AD? (4) Strong evidence was provided that CE treatment extended the life span of prion-infected animals [30–35]. Therefore, it is also worth exploring whether CE administration improves the phenotypic signatures of aging and frailty of mice. (5) The dosage of CE is another point to be considered, as this and other studies used a CE dose of 4 g/kg/SC, which is a very high dose and not applicable to humans, considering an average body weight 70 kg. Therefore, another goal is to improve the bioavailability in the brain through nanoformulation of CEs and determine an applicable dose and route of administration for translational and clinical studies. Once answers to these questions are found, CEs or its modified derivatives can be tested and validated. Potentially, it could then also be of interest as a treatment of other neurodegenerative disorders such as Parkinson’s disease, Huntington’s disease, and tauopathies, as well as in brain disorders associated with systemic inflammation and neuroinflammation.

## Supplementary Information

**Additional file 1: Figure S1**. AβO characterization. **Figure S2**. A representative non-denaturing PAGE of Aβ 6E10. **Figure S3**. Effect of TC-5RW on cell viability of human neuroblastoma N2a cells. **Figure S4**. TC-5RW reduced Aβ amount in the media of SH-SY5Y and N2a cells. **Figure S5**. TC-5RW reduced activated astrocytes and microglia in 5XFAD mice. **Figure S6**. Confocal images of double-immunofluorescence of GFAP (green) and GMFβ (red) staining in the hippocampus regions CA3 and DG of brain. **Figure S7**. Double immunocytochemistry results of GMFβ (green) and GFAP (red) staining in C8D1A astrocytic cells, which were incubated with LPS (1 µg/ml) in the absence and presence of TC-5RW (10 µg/ml) for 24 h. **Figure S8**. Uncropped immunoblotting and dot blotting used in Fig. 3. **Figure S9**. Uncropped immunoblotting results used in Fig. 4. **Figure S10**. Uncropped immunoblotting results in Fig. 5.

## Data Availability

To validate our hypotheses, we presented all the relevant data in this original manuscript file and/or the supplementary information file. Nevertheless, any additional data related to these findings will be provided to the readers upon reasonable request from the corresponding author.
