# Supplementary material for: Cellulose ether treatment inhibits amyloid beta aggregation, neuroinflammation and cognitive deficits in transgenic mouse model of Alzheimer’s disease
Source: J Neuroinflammation. 2023 Jul 28;20:177. doi: 10.1186/s12974-023-02858-y (PMC10375631; doi:10.1186/s12974-023-02858-y)
Supplement: Supplementary file 1 — Additional file 1: Figure S1. AβO characterization. Figure S2. A representative non-denaturing PAGE of Aβ 6E10. Figure S3. Effect of TC-5RW on cell viability of human neuroblastoma N2a cells. Figure S4. TC-5RW reduced Aβ amount in the media of SH-SY5Y and N2a cells. Figure S5. TC-5RW reduced activated astrocytes and microglia in 5XFAD mice. Figure S6. Confocal images of double-immunofluorescence of GFAP (green) and GMFβ (red) staining in the hippocampus regions CA3 and DG of brain. Figure S7. Double immunocytochemistry results of GMFβ (green) and GFAP (red) staining in C8D1A astrocytic cells, which were incubated with LPS (1 µg/ml) in the absence and presence of TC-5RW (10 µg/ml) for 24 h. Figure S8. Uncropped immunoblotting and dot blotting used in Fig. 3. Figure S9. Uncropped immunoblotting results used in Fig. 4. Figure S10. Uncropped immunoblotting results in Fig. 5. [file 12974_2023_2858_MOESM1_ESM.docx]

***J of Neuroinflammation*** *ID 736b76da-317d-487a-93d1-16b5156886b1 R2*

**Cellulose ether treatment inhibits amyloid beta aggregation, neuroinflammation and cognitive deficits in transgenic mouse model of Alzheimer’s disease**

Tahir Ali^1,2*^, Antonia N. Klein^1,2^, Keegan McDonald^1,2^, Lovisa Johansson^3^, Priyanka Ganguli^4^ Mukherjee, Martin Hallbeck^3^, Katsumi Doh-ura^5^, Hermann M. Schatzl^1,2^, Sabine Gilch^1,2*^

^1^ Calgary Prion Research Unit, Faculty of Veterinary Medicine, University of Calgary, Calgary, Alberta, Canada.

^2^ Hotchkiss Brain Institute, University of Calgary, Calgary, Alberta, Canada.

^3^ Department of Biomedical and Clinical Sciences (BKV), Linköping University, 58185 Linköping, Sweden

^4^ Microscopy imaging facility (MIF), University of Calgary, Calgary, Alberta, Canada.

^5^ Department of Neurochemistry, Tohoku University Graduate School of Medicine, Sendai, Miyagi, Japan.

**Running title: Cellulose ethers as repurposed medication for treatment of Alzheimer’s disease**

^*^Corresponding authors

Sabine Gilch, PhD; Associate Professor

Canada Research Chair in Prion Disease Research; Faculty of Veterinary Medicine, University of Calgary, 3330 Hospital Drive NW Calgary, AB T2N 4Z6, Canada.

Office: HRIC1AC66; Phone: + 1 (403) 210-7578; E-mail: [sgilch@ucalgary.ca](mailto:sgilch@ucalgary.ca)

^*^Tahir Ali, PhD

E-mail: [tahir.ali1@ucalgary.ca](mailto:tahir.ali1@ucalgary.ca)


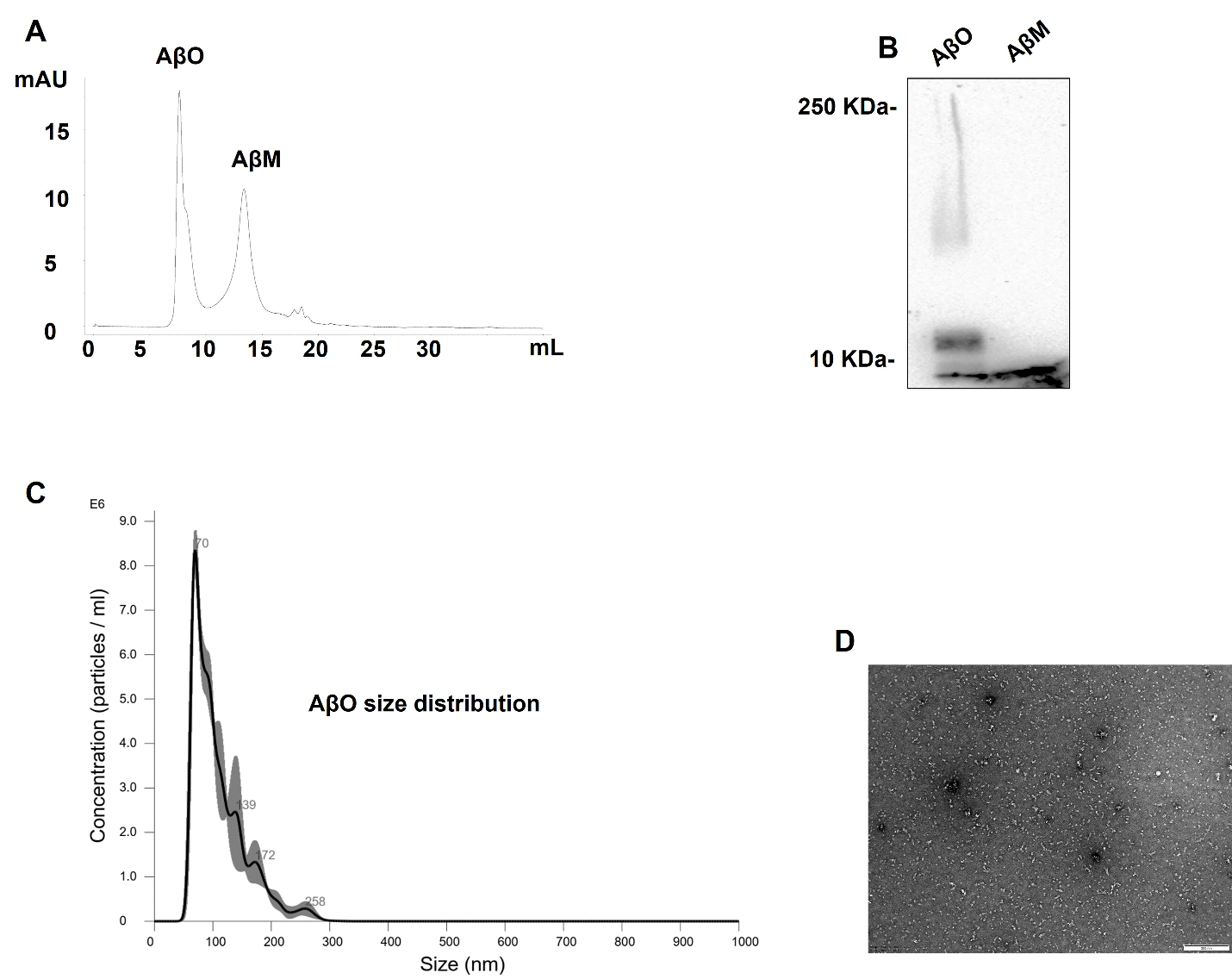


**Fig S1. AβO characterization.** After oligomerization of Aβ, the oligomers (AβO) were separated from monomers (AβM) on a Superdex 75 10/300 GL column by **(A)** collecting the two separate peaks shown in the chromatogram. **(B)** AβO and AβM were run on an SDS page showing higher molecular weight for AβO. **(C)** AβO size was also analyzed using Nanoparticle Tracking Analysis showing a mean size of 109.5nm (+/- 1.3nm SE). **(D)** AβO structure was shown using Transmission Electron Microscopy.


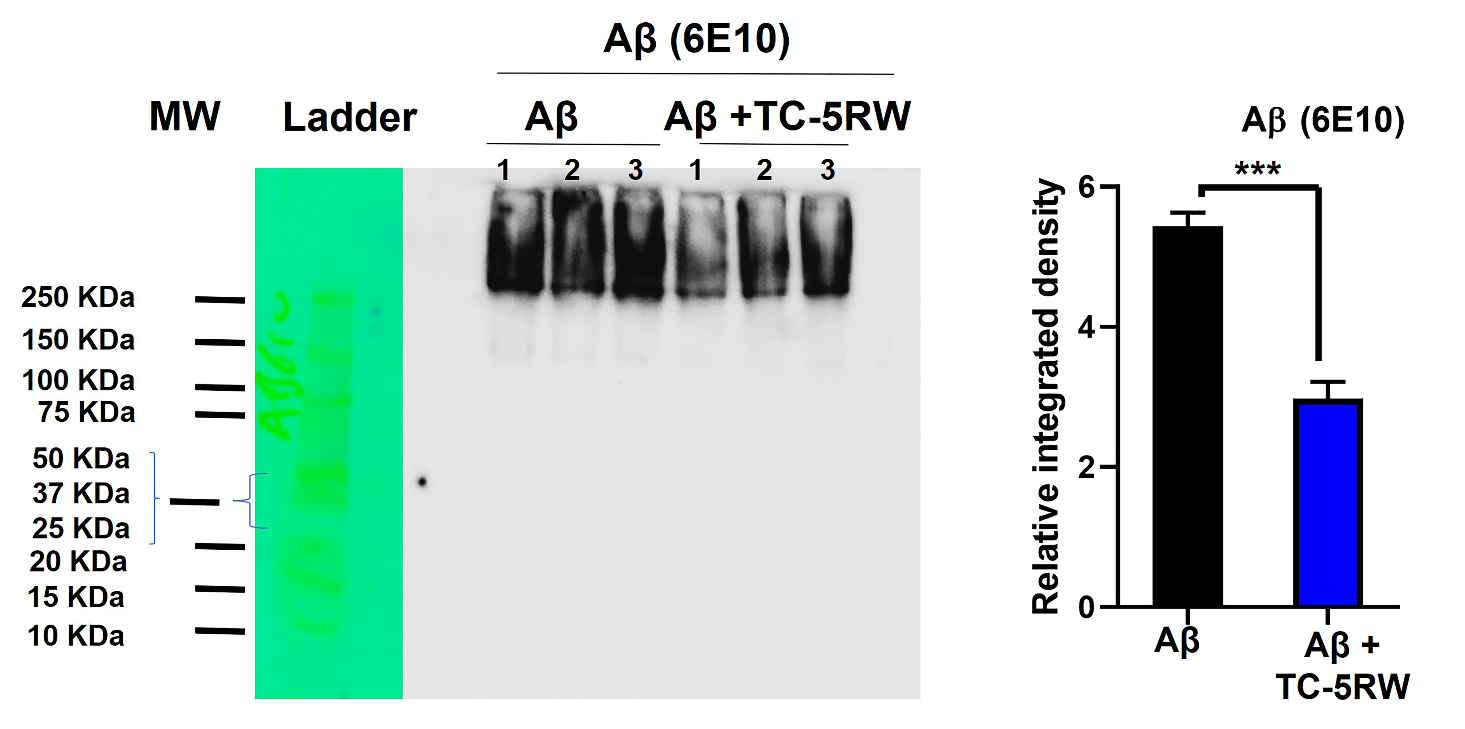


**Fig. 2. A representative nondenaturing PAGE of Aβ 6E10.** Signals in lanes 1, 2, and 3 represent three different tubes for each group. Histogram represents the means ± SEM for the representative signal from three independent experiments. Significance = ***p<0.001.


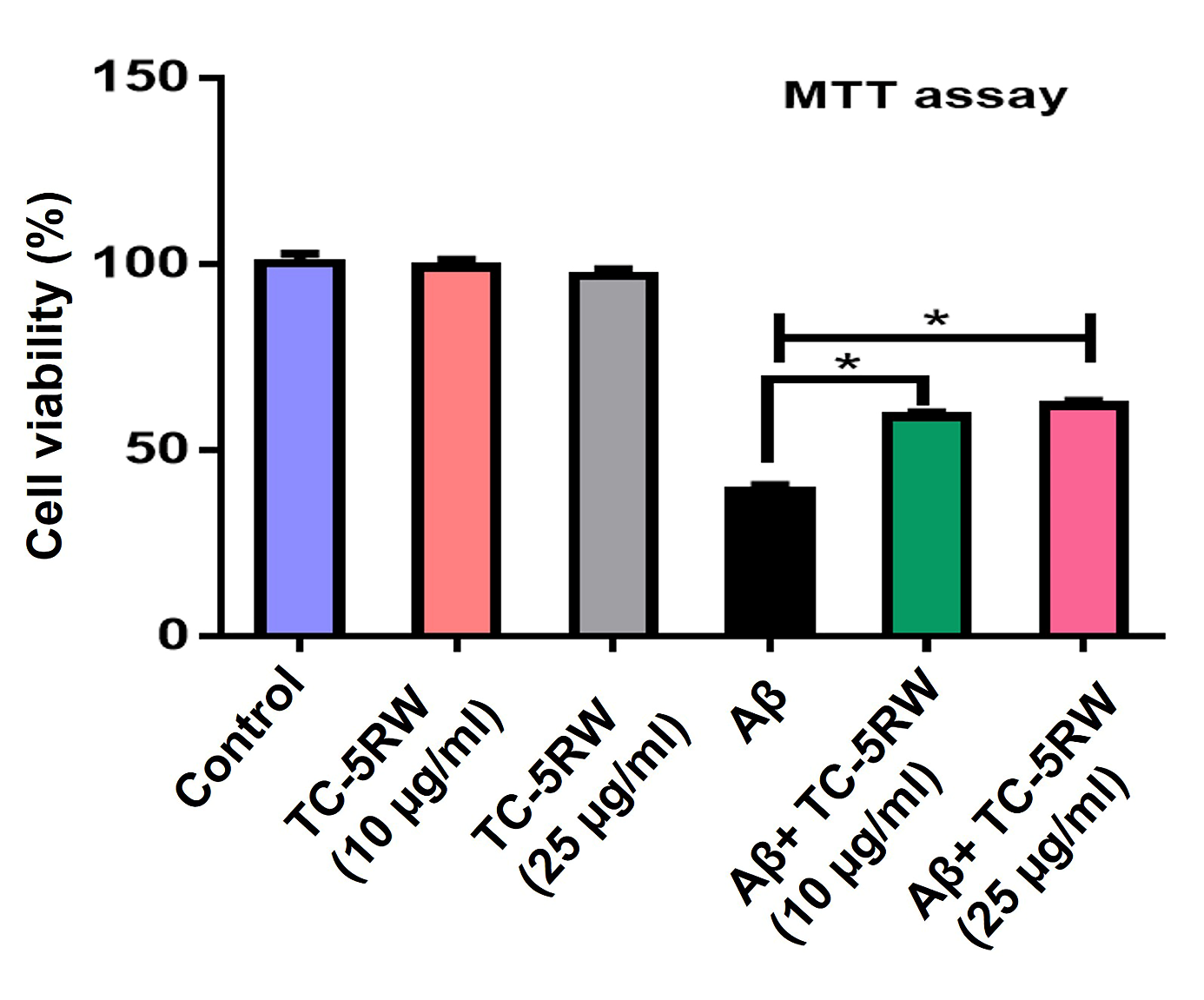


**Fig S3. Effect of TC-5RW on cell viability of human neuroblastoma N2a cells.** MTT assay revealed that Aβ that had been aggregated in the presence of TC-5RW was significantly less toxic to N2a cells than Aβ aggregated without TC-5RW. Aβ aggregates were formed in the absence or presence of TC-5RW (10 µg/ml or 25 µg/ml) *in vitro* and added to the culture medium of N2a cells. After 24 hrs MTT assay was performed. Significance = *p<0.05.


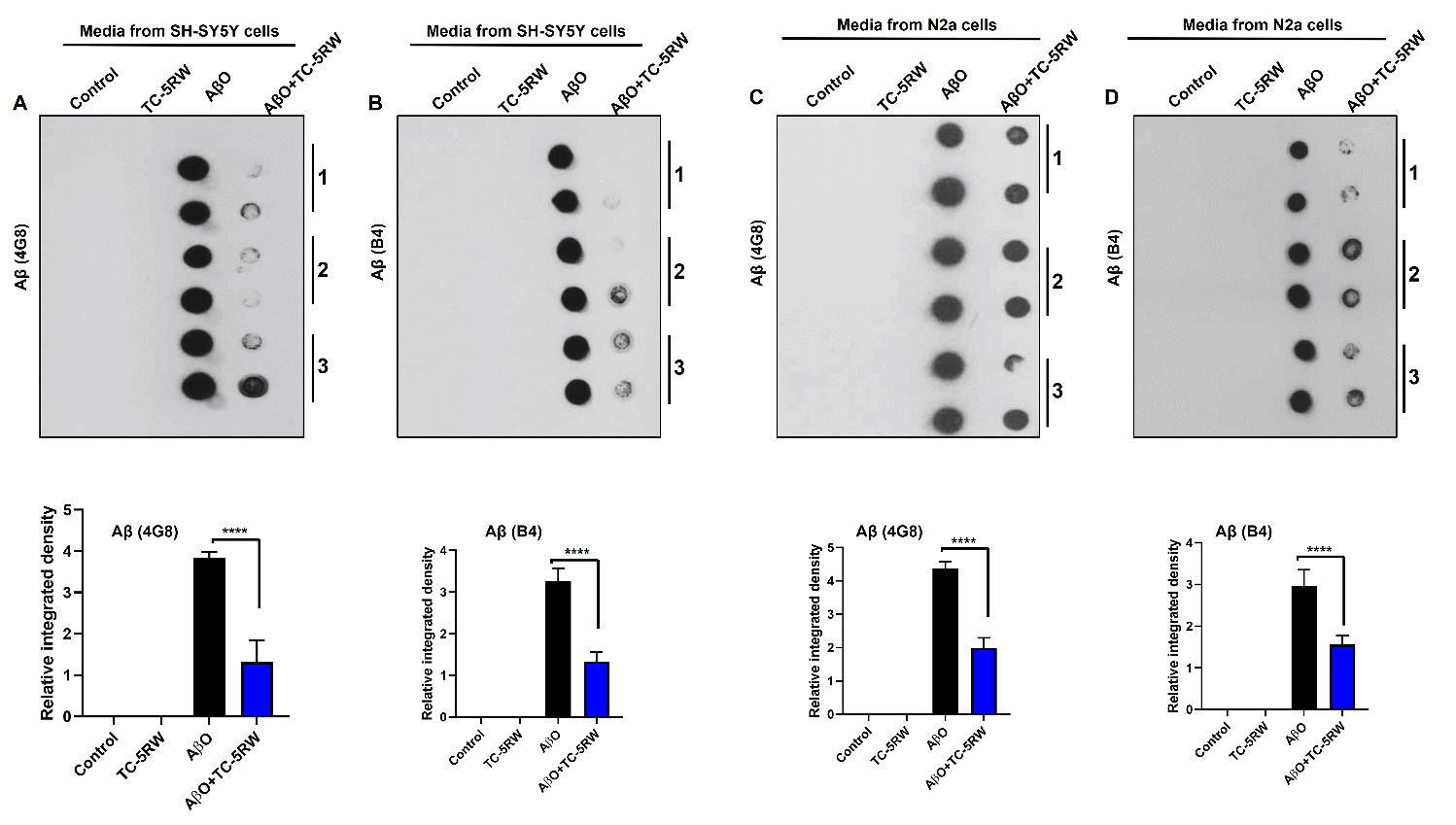


**Fig. S4. TC-5RW reduced Aβ amount in the media of SH-SY5Y and N2a cells. (A-D)** Dot blotting of Aβ (4G8) and Aβ (B4) in the conditioned media of SHSY-5Y and N2a cells that were incubated in AβO (2.5-5µM) in the presence and absence of TC-5RW (10 µg/ml). The dot blot result indicate reduction of Aβ (4G8) and Aβ (B4) levels in the AβO+TC-5RW treated cells as compared to alone AβO treated cells while we did not find any signal in the media of control and TC-5RW alone-treated cells, which confirmed the AβO exposure efficiency and the effect of TC-5RW against AβO. Dots in each groups represent duplicate from the condition media of three independent *in vitro* experiments. Histograms represent the means ± SEM for the representative media of three independent experiments. Significance = **p<0.0001.


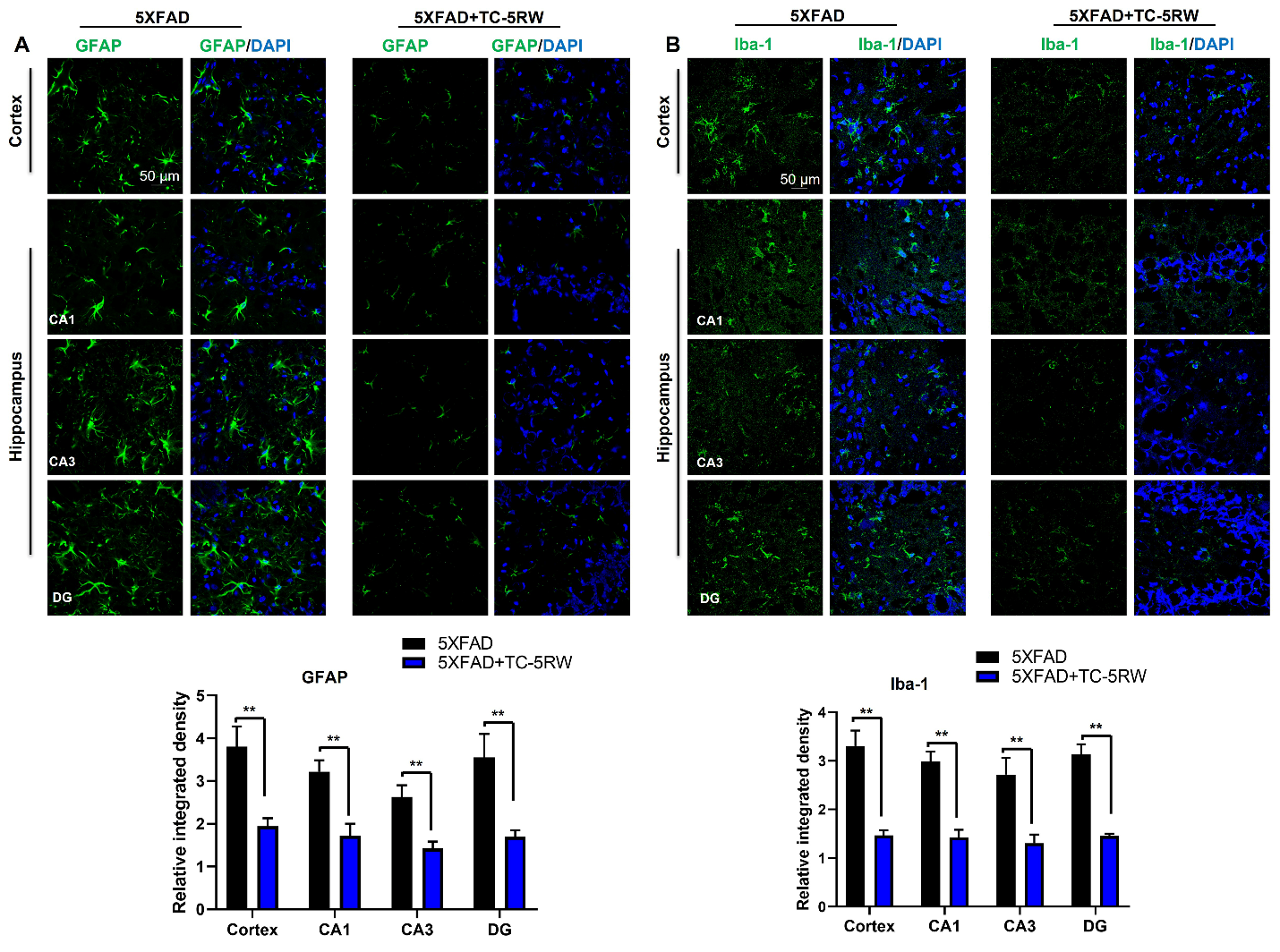


**Fig. S5. TC-5RW reduced activated astrocytes and microglia in 5XFAD mice. (A)** Confocal images of GFAP (green) and DAPI (blue) in the cortex and hippocampus regions (CA1, CA3 and DG) of the brains of non-treated 5XFAD and TC-5RW-treated 5XFAD mice. (**B)** Confocal images of Iba-1 (green) and DAPI (blue) in the cortex and hippocampus regions (CA1, CA3 and DG) of the brains of non-treated 5XFAD and TC-5RW-treated 5XFAD mice. Data are expressed as the means ± SEM for n = 3 mice/group, and the number of independent confocal microscopy experiments = 3. Magnification: 63X. Scale bar = 50 μm. Significance = **p<0.01.


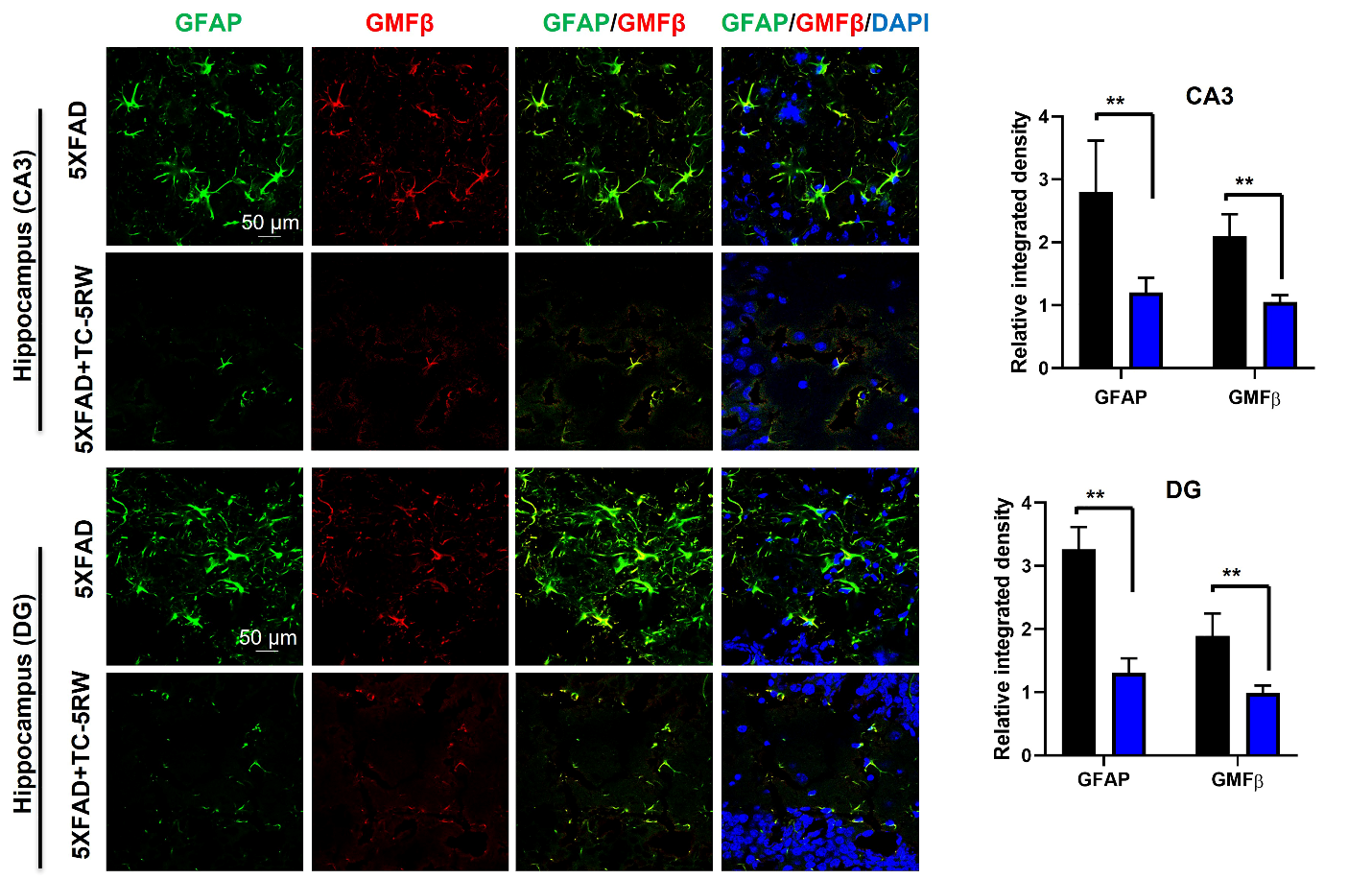


**Fig. S6.** Confocal images of double immunofluorescence of GFAP (green) and GMFβ (red) staining in the hippocampus regions CA3 and DG of brain. Data are expressed as the means ± SEM for n = 3 female mice/group, and the number of independent confocal experiments = 3. Magnification: 63X. Scale bar = 50 μm. Significance = ***p<0.01.


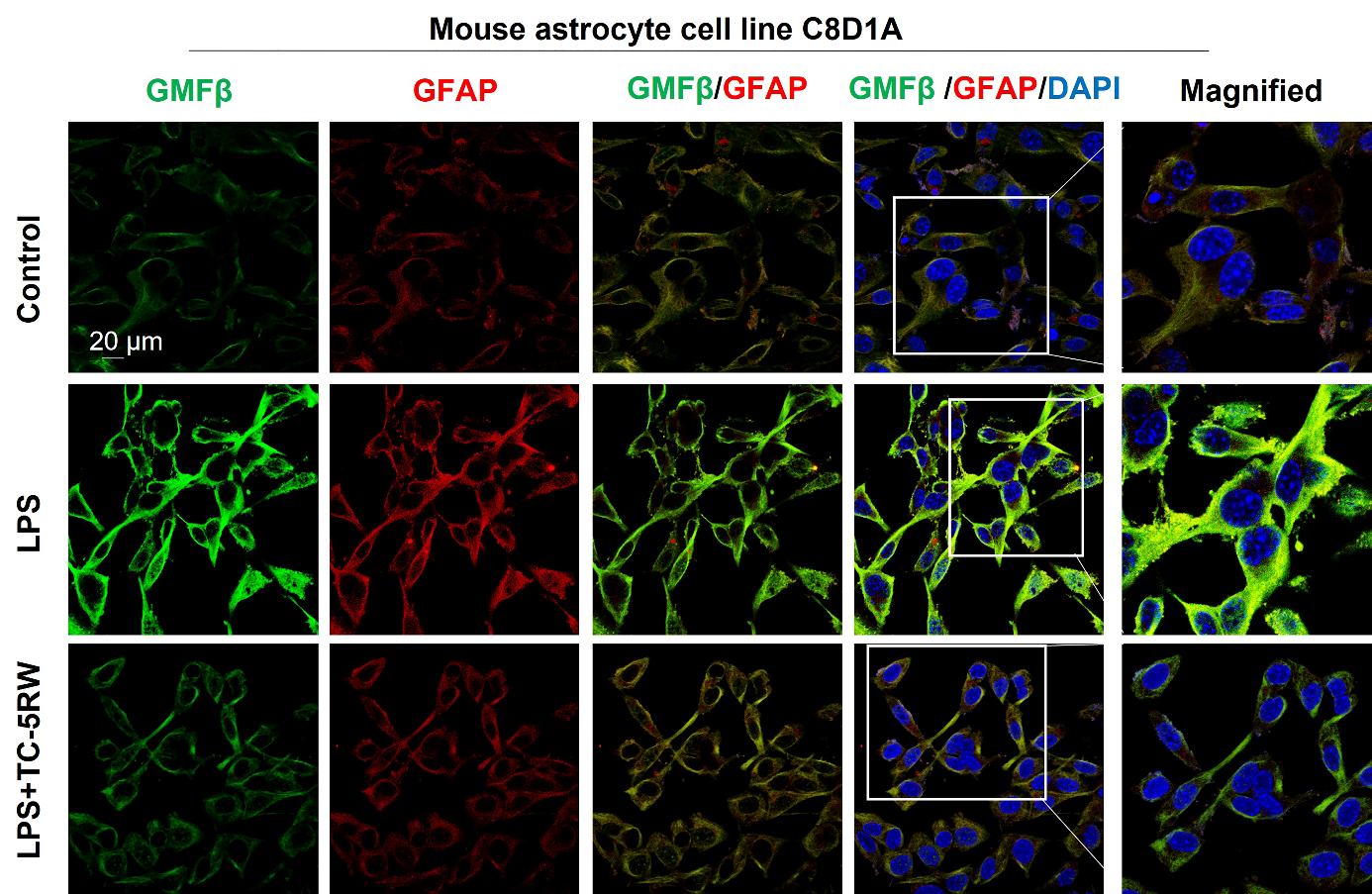


**Fig. S7.** Double immunocytochemistry results of GMFβ (green) and GFAP (red) staining in C8D1A astrocytic cells, which were incubated with LPS (1 µg/ml) in the absence and presence of TC-5RW (10µg/ml) for 24 hrs**.**


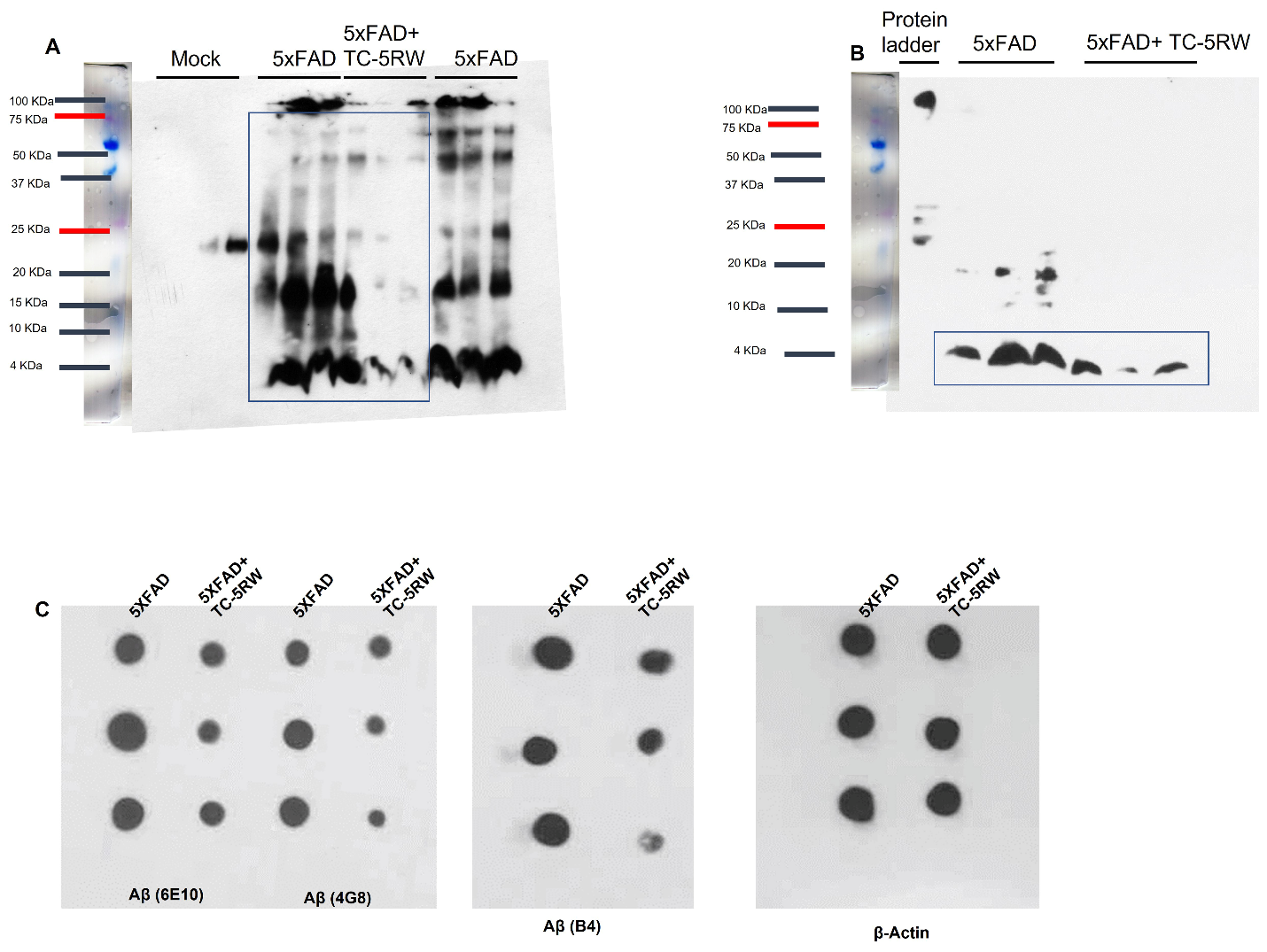


**Fig. S8. Uncropped immunoblotting and dot blotting used in figure 3. (A & B)** Represent the uncropped immunoblotting for Aβ (6E10) antibody for AβO and monomeric level. From panel we cropped and presented the results of 5XFAD and 5XFAD+TC-5RW treated mice. **(C)** Dot blotting uncropped images used in figure 3.


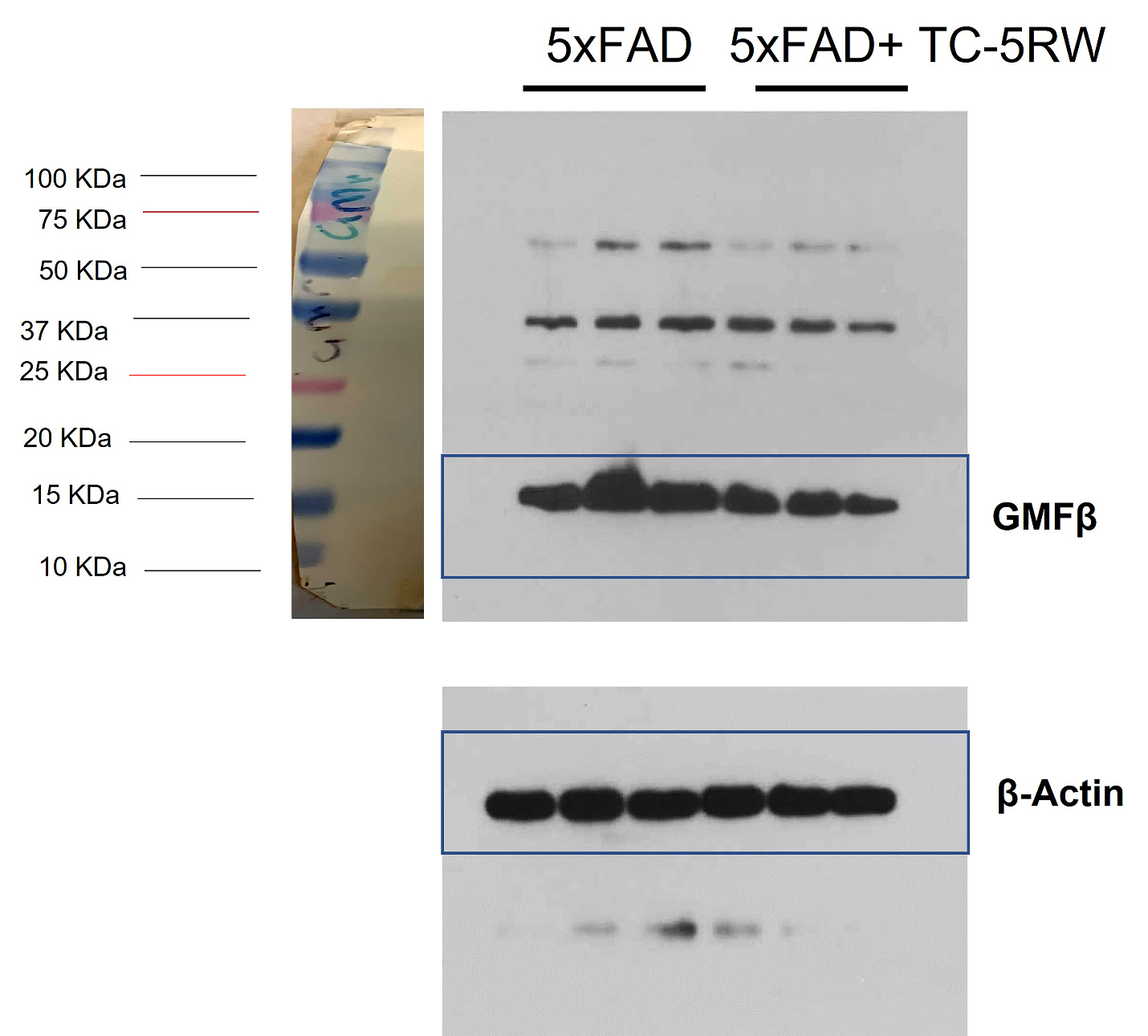


**Fig. S9. Uncropped immunoblotting results used in figure 4.** Represent the uncropped immunoblotting for GMFβ and its corresponding beta actin antibodies.


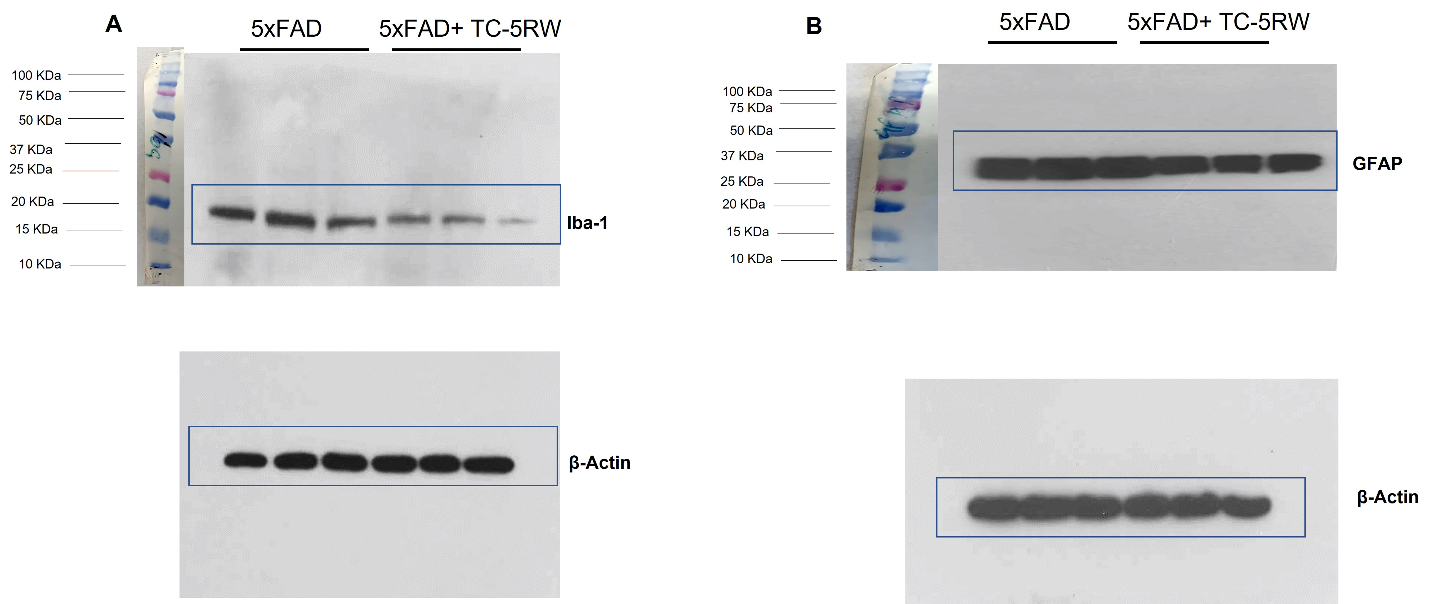


**Fig. S10. Uncropped immunoblotting results in figure 5. (A & B)** Represent the uncropped immunoblotting for Iba-1 and GFAP and their corresponding beta actin antibodies
